# Coexistence of Tellurium Cations and Anions in Phosphonium‐Based Ionic Liquids

**DOI:** 10.1002/chem.202103770

**Published:** 2021-12-28

**Authors:** Matthias A. Grasser, Tobias Pietsch, Jan Blasius, Oldamur Hollóczki, Eike Brunner, Thomas Doert, Michael Ruck

**Affiliations:** ^1^ Faculty of Chemistry and Food Chemistry Technische Universität Dresden 01062 Dresden Germany; ^2^ Mulliken Center for Theoretical Chemistry Institute for Physical and Theoretical Chemistry University of Bonn Beringstr. 4+6 53115 Bonn Germany; ^3^ Department of Physical Chemistry University of Debrecen Egyetem tér 1 H-4032 Debrecen Hungary; ^4^ Max Planck Institute for Chemical Physics of Solids Nöthnitzer Str. 40 01187 Dresden Germany

**Keywords:** ionic liquids, tellurides, tellurium, tellurium cations, ^125^Te NMR spectroscopy

## Abstract

Elemental tellurium readily dissolves in ionic liquids (ILs) based on tetraalkylphosphonium cations even at temperatures below 100 °C. In the case of ILs with acetate, decanoate, or dicyanamide anions, dark red to purple colored solutions form. A study combining NMR, UV‐Vis and Raman spectroscopy revealed the formation of tellurium anions (Te_n_)^2−^ with chain lengths up to at least *n*=5, which are in dynamic equilibrium with each other. Since external influences could be excluded and no evidence of an ionic liquid reaction was found, disproportionation of the tellurium is the only possible dissolution mechanism. Although the spectroscopic detection of tellurium cations in these solutions is difficult, the coexistence of tellurium cations, such as (Te_4_)^2+^ and (Te_6_)^4+^, and tellurium anions could be proven by cyclic voltammetry and electrodeposition experiments. DFT calculations indicate that electrostatic interactions with the ions of the ILs are sufficient to stabilize both types of tellurium ions in solution.

## Graphical abstract

**Grey tellurium dissolves** under disproportionation in the ionic liquids [P_66614_][OAc] and [P_4444_][OAc] at ambient temperatures. The stable red‐purple solutions contain tellurium cations, such as (Te_4_)^2+^ and (Te_6_)^4+^, and anions (Te_*n*_)^2−^ with chain lengths up to at least *n*=5, as evidenced by spectroscopic and electrochemical techniques. DFT calculations indicate that electrostatic interactions with the components of the ILs stabilize tellurium cations and anions in solution.

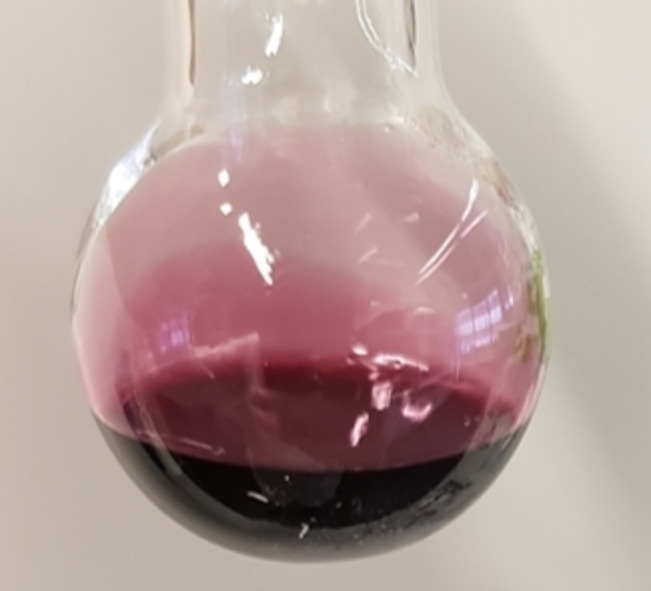

## Introduction

Ionic liquids (ILs) have become a major topic in research and found their way into diverse industrial processes.^[1]^ Several reviews have addressed their advantages as solvents in various fields such as organic synthesis,[^2^, ^3^] inorganic synthesis[^4^, ^5^, ^6^, ^7^, ^8^] or electrochemical application,^[9]^ to name a few. The growing importance of ILs stems from their great diversity and adaptability for specific tasks, with properties that may include almost negligible vapor pressure, a wide liquid range, high thermal and chemical stability, and wide electrochemical window.

Aromatic nitrogen‐based cations such as imidazolium or pyridinium are the most studied group of ILs.^[10]^ They are often preferred for inorganic synthesis because of their good solvation properties, which are tunable by modification of the cation and variation of the anion.^[11]^ Meanwhile, phosphonium‐based ILs are gaining interest because of their higher temperature stability and reduced C−H acidity compared to imidazolium ILs.[^10^, ^12^, ^13^, ^14^] Phosphonium acetate ionic liquids are discussed as solvent and additive for biomass processing and for CO_2_ capture.[^15^, ^16^, ^17^, ^18^] Additionally carboxylate phosphonium ILs are discussed in applications as heat transfer fluids.[^12^, ^19^]

It has already been shown that phosphonium ILs are capable of dissolving various elements to form ionic species. Boros et al. observed a red solution when red phosphorous was treated with trihexyl(tetradecyl)phosphonium decanoate [P_66614_][dec].^[20]^ Wolff et al. studied dissolution of red phosphorus in the acetate [P_66614_][OAc] and identified (P_16_)^2−^ as an ionic species in solution.^[21]^ However, no associated phosphorus cation was identified in either system. Boros et al. also demonstrated the solubility of chalcogens in [P_66614_][dec]. They observed a blue colored solution after the dissolution of S_8_ in the IL which was attributed to the formation of the radical anion (S_3_)^.−^. At lower temperatures, the mixture turns red because the equilibrium of (S_3_)^.−^ with (S_6_)^2−^ shifts to the side of the dimer. Selenium and tellurium were also dissolved in this IL, resulting in an orange and deep purple color, respectively. The presence of (Se_3_)^.−^ was suspected, while the dissolved tellurium species remained unknown.^[20]^

Zhang et al. showed that one of the activation mechanisms of tellurium in phosphonium ILs, especially at temperatures above 200 °C, is based on the formation of trialkylphosphane tellurides R_3_P−Te, which lead to the formation of yellow solutions. The method can be used for the controlled synthesis of tellurium nanoparticles but includes the decomposition of the IL.[^22^, ^23^]

Below 100 °C, the activation of tellurium in phosphonium ILs proceeded without the formation of R_3_P−Te and thus preserving the IL.^[24]^ Stable red solutions formed with the acetate [P_66614_][OAc] and the dicyanamide [P_66614_][DCA], similar to what had been described for [P_66614_][dec] by Boros et al. In [P_66614_]Cl, subsequent reaction of the solution with silver or gold yielded the respective metal tellurides.^[24]^ These results indicate a temperature‐ and anion‐specific behavior of phosphonium ILs, especially when the anion can be considered as a base. The color of the solutions suggests the formation of oligotellurides in the IL.^[25]^ However, the formation of anions requires either a reducing agent (possibly the IL) or the simultaneous formation of tellurium cations.

Ditelluride (Te_2_)^2−^ and tritelluride (Te_3_)^2−^ anions were originally synthesized by the reaction of elemental tellurium with alkali metals in liquid ammonia.[^26^, ^27^, ^28^] In DMF or liquid ammonia, the mono‐, di‐ or tritelluride can react with elemental tellurium to form tetratelluride (Te_4_)^2−^.[^25^, ^29^] Haushalter et al. used an aqueous solution of tetrabutylammonium bromide [Bu_4_N]Br and K_2_Te_3_ to synthesize [Bu_4_N]_2_(Te_5_) from a dark purple solution. They assumed a simultaneous formation of (Te_n_)^2−^ with n <3. (Te_5_)^2−^ also proved to be stable in acetone.^[30]^ Albrecht et al. used As_2_O_3_ to reduce TeO_2_ and SeO_2_ in ultra‐alkaline aqueous KOH solution and obtained K_2_Te_3_, K_2_Se_3_ and K_2_TeSe_2_, although only the mono‐ and dichalcogenides were detected in the solution.^[31]^

Homoatomic tellurium cations have been isolated in several compounds which were crystallized from different ILs.^[32]^ (Te_6_)^2+^ in (Te_6_)[WOCl_4_]_2_ was synthesized in a mixture of [BMIm]Cl and AlCl_3_ by oxidation of elemental tellurium with WOCl_4_.^[33]^ The cations (Te_4_)^2+^ and (Te_8_)^2+^ are accessible via comproportionation of tellurium and TeCl_4_ in the presence of BiCl_3_ in Lewis‐acidic ILs [BMIm]Cl ⋅ nAlCl_3_ (*n*=1.3–1.5). Te_4_[Bi_0.74_Cl_4_], Te_4_[Bi_6_Cl_20_], and Te_8_[Bi_4_Cl_14_] were isolated from these systems.[^34^, ^35^] Without BiCl_3_, the tetratellurium cation precipitated in the salt Te_4_[AlCl_4_]_2_.^[33]^ Similarly, tellurium and TeI_4_ in the presence of red phosphorous yielded Te_4_[Al_2_Cl_7_].^[35]^ Feldmann et al. crystallized (Te_8_)_2_[Ta_4_O_4_Cl_16_] starting from Te, TeCl_4_, TaOCl_3_ and TaCl_5_ in [BMIm]Cl.^[36]^ Beck et al. used an electrochemical approach to synthesize (Te_4_)^2+^, (Te_6_)^4+^ and (Te_8_)^2+^ by the oxidation of tellurium in tetraalkylammonium and imidazolium ILs and isolated Te_4_[CTf_3_]_2_, Te_6_[OTf]_4_ and Te_8_[NTf_2_]_2_.^[37]^

To date, there is little information on the tellurium species present in ILs at moderate temperatures, thus an important element in understanding the reactivity of tellurium in ILs is missing. We have therefore performed extensive studies on the dissolution of tellurium in phosphonium ILs, particularly in the room temperature IL (RTIL) [P_66614_][OAc], at temperatures up to 100 °C. In particular, we have investigated the question of whether (polyatomic) tellurium cations and anions coexist in the IL.

## Results and Discussion

### Observation: Tellurium dissolves in phosphonium ILs at 100 °C

When testing various phosphonium‐based ionic liquids for the synthesis of silver and gold tellurides, starting from the elements, some ionic liquids containing the anions [DCA]^−^, [dec]^−^ or [OAc]^−^ showed insufficient reaction to tellurides but strong red coloration of the liquid phase.^[24]^ After addition of EtOH, DCM or THF, the solution turned into a brownish‐black suspension of tellurium particles. The red reaction mixtures of ionic liquids were found to be stable over the long term under inert conditions after all solid components had been separated (see Figure S1 in Supporting Information). In the case of the decanoate IL, its high viscosity and gelation, which already occurs at room temperature, are problematic for the formation of long‐term stable solutions.

In further investigation of these systems, we have tried to avoid the influence of impurities, which are common in phosphonium‐based ILs. We developed two purification protocols for [P_66614_][OAc], and used the purified IL for further investigations. The first way is the purification of [P_66614_]Cl by column chromatography followed by anion exchange with K[OAc] according to a literature procedure.^[24]^ In the second path, the anion exchange was performed prior to the chromatographic purification of the target IL. In view of the difficulty and low yield of the purification of [P_66614_][OAc], we repeated the dissolution experiment with tetrabutylphosphonium acetate [P_4444_][OAc]. This IL was synthesized in the same way as [P_66614_][OAc] by metathesis from [P_4444_]Cl with K[OAc].^[24]^ The purity of commercial [P_4444_]Cl is much higher compared to [P_66614_]Cl. In particular, the amount of trialkylphosphane or HCl impurities is reduced, eliminating the need for additional purification. [P_4444_]^+^ is the smallest tetraalkylphosphonium cation that forms ILs by definition, and therefore also serves as a model system for theoretical investigations. However, [P_4444_][OAc] is solid at room temperature and the deep red solution formed at 100 °C solidifies after cooling to room temperature, too. Given the higher solubility of tellurium in [P_66614_][OAc] and the analytical preference of liquid samples at room temperature, we chose [P_66614_][OAc] for the following investigations. Reported decomposition temperatures, determined by thermogravimetric analysis (TGA), are far higher than the applied dissolution temperatures.[^12^, ^38^]

As mentioned above, red colored solutions have been known in tellurium chemistry for a long time. On the one hand, the red coloration of concentrated H_2_SO_4_, oleum, or HSO_3_F after dissolution of elemental tellurium is due to the oxidation of tellurium to (Te_4_)^2+^.^[39]^ On the other hand, deep red and violet colored solutions of oligotellurides from (Te_2_)^2−^ to (Te_5_)^2−^, which can be synthesized by reduction of tellurium with alkali metals in an alkaline solvent, are also known.[^28^, ^30^, ^40^]

In contrast, elemental tellurium dissolves in [P_66614_][OAc] or [P_4444_][OAc] without any sign of a chemical reaction with the IL, suggesting disproportionation of tellurium.

### Liquid‐state NMR spectroscopy: Dissolved tellurium molecules and no reaction of the IL

We investigated the tellurium‐containing IL solutions by ^31^P and ^125^Te NMR spectroscopy. ^31^P with a natural abundance of 100 % and a nuclear spin quantum number of *I*=1/2 renders ^31^P NMR spectroscopy highly suitable for monitoring the stability of the phosphonium IL. In addition, ^125^Te NMR spectroscopy can be used for gaining information on dissolved tellurium species. However, it suffers from the low natural abundance of the NMR‐sensitive tellurium isotope ^125^Te (7.07 %). This is particularly problematic because the concentrations of dissolved species are low. To overcome this problem, we have performed experiments with 99.7 %‐enriched ^125^Te. The solutions were handled under inert conditions in NMR tubes with a PTFE valve seal, as they are sensitive to air and moisture. The deuterated solvent was kept separate from the sample in a sealed capillary, since dilution of the sample with another solvent immediately leads to precipitation of tellurium, as has been previously reported for solutions of tellurium in [P_66614_]Cl.[^22^, ^24^]

Using dried, unpurified [P_66614_][OAc] and tellurium with natural isotope distribution at 100 °C, we did not observe a tellurium signal. With ^125^Te enriched tellurium, however, we detected two doublets at −904.5 ppm and −837 ppm with coupling constants of *J*(^31^P,^125^Te)=1711 Hz and *J*(^31^P,^125^Te)=1694 Hz, respectively (Supporting Information Figure S2). Doublets with identical coupling constants were also found in the ^31^P NMR spectra, indicating that tellurium is bound to phosphorus (Supporting Information Figure S3). These signals were known from previous works and can be assigned to products of the reaction of tellurium with trialkylphosphane (P*R*_3_) impurities in the IL.[^24^, ^41^, ^42^] Another doublet is located at −510 ppm and exhibits a coupling constant of *J*(^31^P,^125^Te)=1460 Hz. Similar to the previously discussed signals, a corresponding doublet is found in the ^31^P NMR spectrum at 14 ppm. Based on the coupling to phosphorus and the magnitude of the *J*(^31^P,^125^Te) coupling constant, it is very likely that this signal originates from another organophosphorus(V) telluride formed by the reaction of tellurium with phosphane impurities.^[42]^

**Figure 1:**
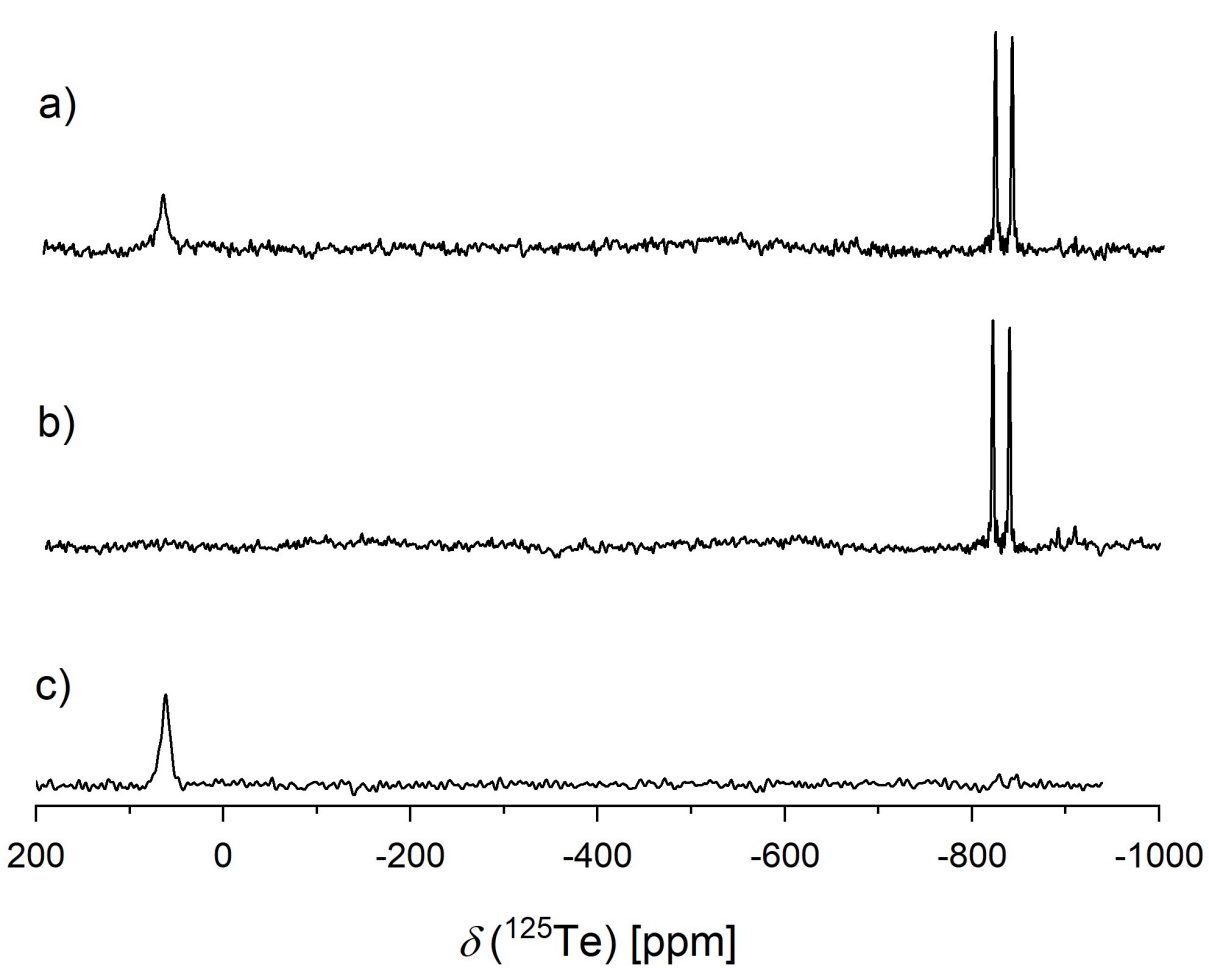
^125^Te NMR spectra of elemental tellurium dissolved in unpurified [P_66614_][OAc] in inert atmosphere (a), the same sample after exposure to air (b) and elemental tellurium dissolved in purified [P_66614_][OAc], which shows no doublet of R_3_P−Te species (c).

In contrast to the described doublets with narrow lines at negative chemical shift, a third signal observed at about 60 ppm is rather broad (Figure 1a). The absence of a *J*‐coupling and the lack of a corresponding signal in the ^31^P spectrum indicate a dissolved tellurium species that is not bound to phosphorous. In air, the deep red color disappears, followed by precipitation of a dark powder, which was identified as elemental tellurium by PXRD. The signal at 60 ppm disappears from the ^125^Te NMR spectrum of the decolorized solution after air contact, whereas the signals of the organophosphorus(V) telluride are still present (Figure 1b).

These observations prove that the red color of the IL solution originates from a phosphorus‐free tellurium species. This species precipitate to elemental tellurium after air contact of the IL solution.

The ^31^P NMR measurements did not show any evidence for a decomposition of the IL during the solvation at temperatures up to 100 °C. We repeated the experiments with purified IL.^[18]^ The ^31^P NMR spectrum of purified [P_66614_][OAc] does not exhibit the above‐described signals of trialkylphosphane impurities. When the purified IL was used to dissolve tellurium, the signal at 60 ppm again occurs in the ^125^Te NMR spectrum and the signals of trialkylphosphane tellurides are absent (Figure 1c). We also used the IL [P_4444_][OAc], which contains a smaller cation and is thus more suitable for quantum chemical calculations. The NMR spectra were recorded at 70 °C to exceed the melting point of the IL at 58 °C. The ^31^P NMR spectrum showed no evidence of phosphane impurities (Supporting Information S3). The broad signal in the ^125^Te NMR spectrum was shifted to 69 ppm (Supporting Information Figure S2). The results show, that neither phosphane impurities nor a reaction of the IL is involved in the formation of the dissolved species. This suggests that the signal at 60 ppm corresponds either to a neutral tellurium species or to an ionic tellurium species that forms also without the presence of P*R*_3_ impurities. A plausible explanation would be a disproportionation leading to soluble cationic and anionic tellurium species and a comproportionation to the element during precipitation. The color of the solution indicates ionic tellurium species but this implies that NMR experiments did not detect all dissolved species.

Colored tellurium ions are the oligotellurides (Te_n_)^2−^ with n=2–5 and cations such as (Te_4_)^2+^ or (Te_6_)^4+^.[^29^, ^40^, ^43^, ^44^] Björgvinsson and Schrobilgen measured NMR spectra of oligotellurides (n=2–4) in ethylenediamine (en) and liquid ammonia. They found that the chemical shift increases with the length of the tellurium chain. The highest chemical shift of 19 ppm was reported for (Te_4_)^2−^.^[29]^ Following this trend, (Te_5_)^2−^ should have an even higher chemical shift. They additionally concluded that NMR investigations of longer chains should be affected by intra‐ and intermolecular exchange of tellurium atoms. Consequently, the signals of the tellurium atoms should be broadened and averaged to one signal even if more signals for chemically different nuclei are expected. This could be the reason why we observed only one broad signal in the ^125^Te NMR spectrum, although the oligotellurides (Te_4_)^2−^ and (Te_5_)^2−^ should generate two and three multiplets, respectively. Another reason might be the interaction of the terminal Te atom with other species in the IL solution leading to a broadening and consequently suppression of signal assigned to this terminal atoms.

Lowering the temperature could slow down the exchange, to a rate that can be considered “slow” on the time scale of ^125^Te NMR measurements. At this point, separate signals should be visible for the chemically distinct nuclei. However, the viscosity of the ionic liquid increases considerably upon cooling, which can lead to a broadening of the signals, too. The effect of temperature on the signal at 60 ppm between −40 °C and 80 °C was investigated. Lowering the temperature led to a broadening of the signal along with a decrease in intensity until disappearance; no new signals appeared. When the solution was heated, the signal became narrower up to about 45 °C, indicating this value as optimum measurement temperature. At higher temperatures, the signal broadened and the signal‐to‐noise ratio decreases again (Figure S4). For an averaged signal under “fast” exchange, further narrowing is expected at increasing temperatures until the line reaches a final narrow line shape. Since this is not observed for the studied signal, we assume that the signal corresponds to a single tellurium species. Based on the broad, temperature‐dependent signal, we can conclude that the investigated tellurium species interacts with other species, for example by ion pair formation, and/or undergoes internal conformational changes. The fact that only a singlet and no *J*‐coupling was observed suggests a ring or polyhedral structure common for cationic tellurium molecules.[^36^, ^43^, ^44^, ^45^, ^46^] For the cations (Te_4_)^2+^ and (Te_8_)^2+^ chemical shifts of about 2700 ppm are reported in the literature.[^37^, ^43^] We observed a similar value for (Te_4_)^2+^ that was synthesized by oxidizing elemental Te in H_2_SO_4_ (Supporting Information Figure S5). Thus it is very unlikely that the signal observed in the IL solution corresponds to this cations. For (Te_6_)^4+^ in 30 % oleum a chemical shift around 150 ppm was reported, which is closer to the observed signal of the Te species dissolved in the ionic liquids but cannot confirm this cation beyond doubt.^[43]^ The solvent have probably a strong influence on the chemical shifts.

Further structural information on the solute tellurium species is not provided by NMR spectroscopy for the discussed reasons. No other signals appeared in the spectra between −1200 ppm and 3000 ppm. It is likely that we did not observe signals from all solute species because NMR may be “blind” for some dissolved species in the reaction mixture. In addition to the dynamic process discussed above, an NMR signal could be broadened due to the size of the species and its restricted mobility. Another reason could be a variety of structurally different tellurium species at low concentrations (i. e., structural inhomogeneity).

In summary, the NMR measurements confirm the dissolution of tellurium in [P_66614_][OAc] and [P_4444_][OAc] independent of the presence of trialkylphosphane impurities and with preservation of the ILs. To gain more information about the tellurium species in solution additional techniques were applied.

### UV‐Vis absorption spectroscopy: Identification of anions (Te_n_)^2−^ with 1≤*n*≤5

**Figure 2:**
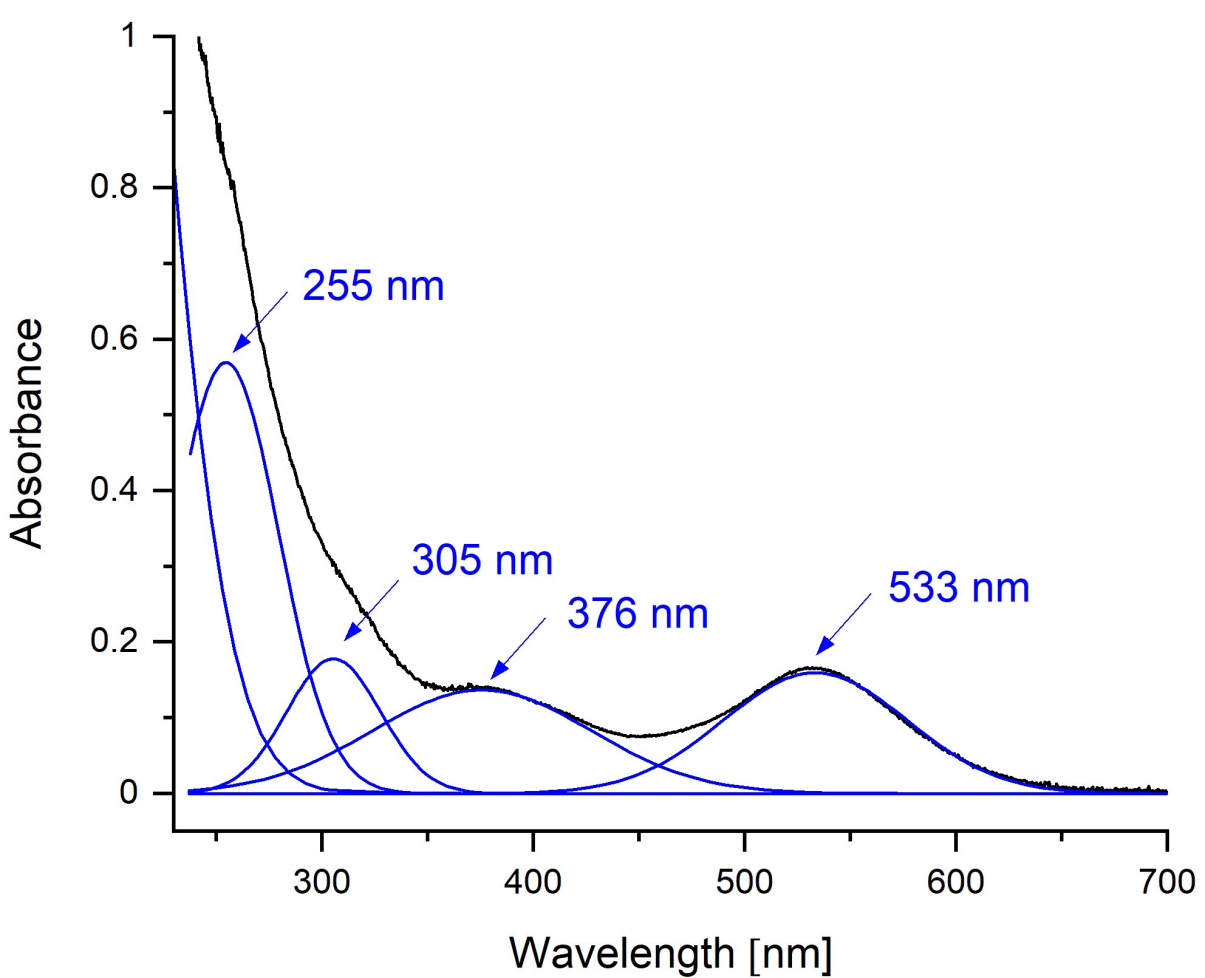
Absorption spectrum of a tellurium solution in [P_66614_][OAc] (black) with peak fitting at 533, 376, 305 and 255 nm (blue).

Diluting the intensely colored solutions with a solvent other than the IL resulted in immediate precipitation of elemental tellurium. To solve this problem, we used fused silica cuvettes with an optical path length of 1 mm. The cuvettes containing the samples were prepared in the glovebox, transported to the spectrometer in a closed container, and measured immediately. Fitting the absorption spectrum of a solution of tellurium in [P_66614_][OAc] revealed absorption maxima at 533, 376, 305 and 255 nm (Figure 2). Strong absorption below 250 nm made detection of additional signals difficult. Haushalter et al. assigned the absorption bands at 375 and 530 nm observed for telluride dissolved in acetone to (Te_5_)^2−^.^[30]^ Myers found a strong absorption in the UV below 260 nm for (Te_2_)^2−^ in aqueous solution and another weaker local absorption maximum at 508 nm while (Te)^2−^ absorb at 325 nm and 280 nm.^[40]^ McAfee et al. assigned an absorption at 530 nm to (Te_4_)^2−^ and at 376 nm to (Te_3_)^2−^ in DMF solution.^[25]^ Thus, the observed UV‐Vis spectrum indicates that tellurides of various chain lengths are present in the IL after dissolution of tellurium.

As a reference, we used K_2_Te_3_ freshly prepared with the hydroflux method according to Albrecht et al.^[25]^ and dissolved it in [P_66614_][OAc]. In the aqueous KOH solution used for the preparation for K_2_Te_3_, Albrecht et al. only identified Te^2−^ and (Te_2_)^2−^ by their absorption bands at 324 nm and 522 nm, respectively.^[31]^ The spectrum of [P_66614_][OAc] after dissolution of K_2_Te_3_ showed the same multiple features as the spectrum after dissolution of elemental tellurium. Obviously, an equilibrium between the different tellurides (Te_n_)^2−^ is established in the IL (Eq. 1).
(1)

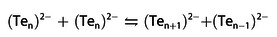




To gain insight into the mechanism, we performed the dissolution of tellurium at only 50 °C in the glovebox and measured UV‐Vis absorption spectra at different reaction times (Supporting Information S6). Immediately after the start of the reaction, a very strong absorption below 300 nm obscures detailed information in this region. 30 minutes after the start of the reaction, an absorption appeared at 530 nm. After 90 minutes, both absorptions at 530 and 375 nm were detectable and increased in intensity as the reaction time progressed.

To access the absorptions at higher energies, the experiment was repeated with a drop of the sample prepared as a thin layer between two sapphire glass plates. Using this method, we were able to resolve another absorption maximum at 220 nm that occurred immediately after the reaction began. After 40 minutes, a shoulder appeared at 250 nm. According to Myers, (Te)^2−^ and (Te_2_)^2−^ exhibit absorption maxima in this range.^[40]^ This suggest that monotelluride formed first, which then reacted with either solid tellurium or dissolved tellurium species to oligotellurides.

Although the disproportionation of tellurium must also give rise to cationic tellurium species, it is impossible to undoubtedly detect them in the UV‐Vis spectra because the expected absorption maxima of (Te_4_)^2+^ at 520 nm and (Te_6_)^4+^ at 250 nm overlap with the absorption of the tellurides.^[45]^ However, the presence of multiple tellurides confirms that charged tellurium species form during the solvation of the element.

Unfortunately, it was not possible to characterize the dissolved tellurium species by mass spectroscopy. None of the organic solvents tested (DCM, EtOH, toluene), which were needed to dilute the solution, were suitable. In all cases, the solution decomposed upon addition of the solvent with precipitation of tellurium.

### Raman spectroscopy: Evidence for (Te_n_)^2−^ with *n*>3

**Figure 3:**
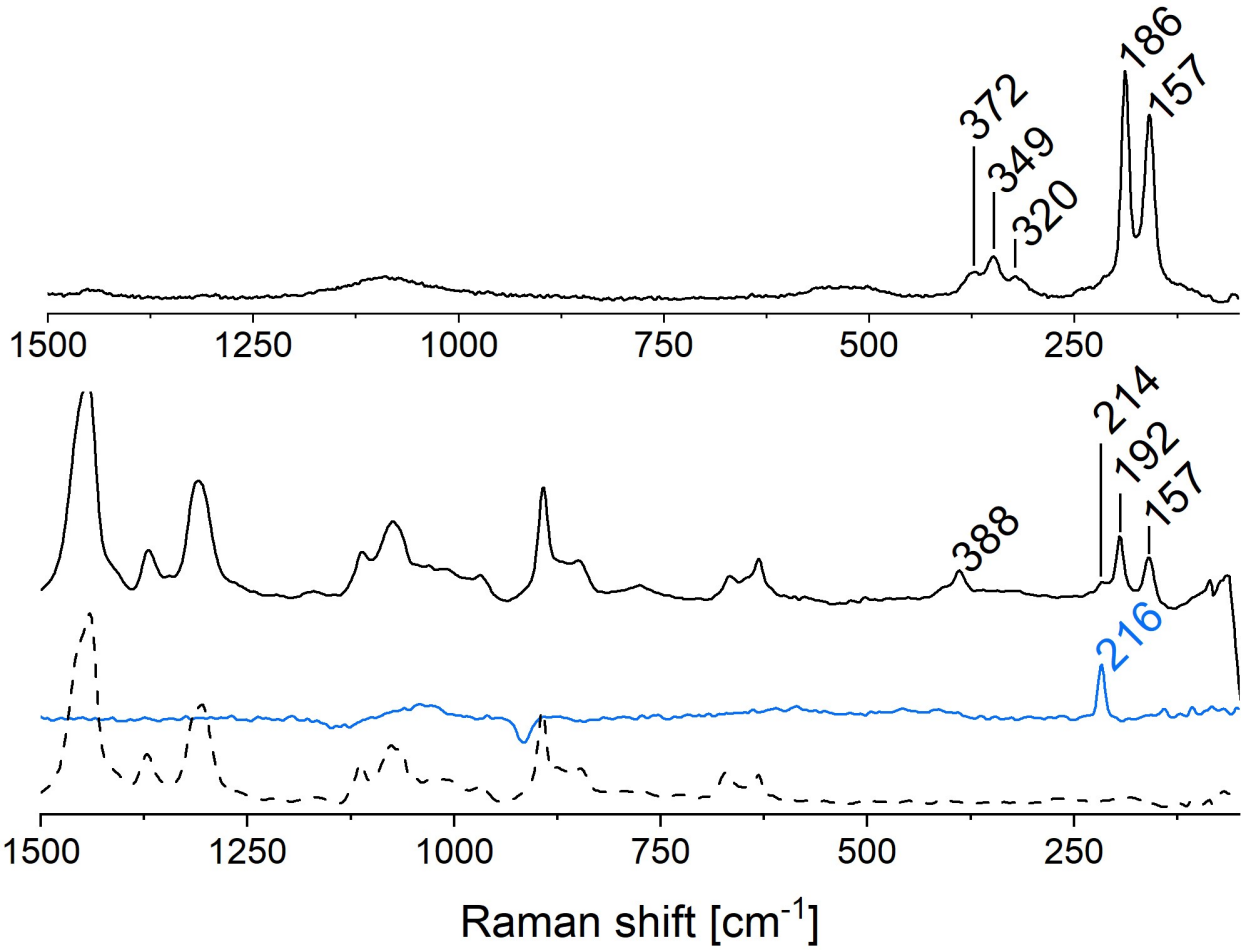
Raman spectra of Te dissolved in [P_66614_][OAc] measured with a 532 nm (top) and 1064 nm (bottom) excitation laser. The dashed line represents the spectrum of neat [P_66614_][OAc] measured with a 1064 nm excitation laser. The blue line represents (Te_4_)^2+^ prepared by dissolving elemental Te in hot H_2_SO_4_. The Raman spectrum of H_2_SO_4_ was subtracted from this spectrum. All spectra are baseline corrected. A polynomial fluorescence correction was applied to the 532 nm spectrum.

Raman spectroscopy with excitation wavelengths of 1064 nm or 532 nm was applied to study the purple‐red solutions. Using the 1064 nm laser, we observed two bands at 192 and 157 cm^−1^ (Figure 3). Both are known for Te–Te stretching vibrations of tellurides.[^30^, ^47^] The stretching modes of (Te_2_)^2−^ and (Te_3_)^2−^ are located around 160 cm^−1^.^[25]^ Haushalter et al. observed Raman signals at 195 cm ^−1^ and 170 cm^−1^ for (Te_5_)^2−^.^[30]^ The observed bands indicate that in addition to di– and tritelluride, tellurides (Te_n_)^2−^ with chain lengths *n* >3 are also present in the IL solution.

Next to the band at 192 cm^−1^, the weak shoulder at 216 cm^−1^ might be a hint for the presence of the aromatic cation (Te_4_)^2+^ (Figure 3). Another band at 388 cm^−1^, which is absent in the Raman spectrum of untreated [P_66614_][OAc] (dashed line), can be assigned to organophosphorus(V) tellurides.^[48]^ Using a 532 nm laser, fluorescence overlaid almost the entire spectrum. Most of the signals from the IL cations and anions are therefore not visible in the fluorescence‐corrected spectrum. However, the two signals of the Te–Te stretching vibrations are still visible, with the band at 192 cm^−1^ shifted to 186 cm^−1^. Additional bands between 380 and 320 cm^−1^ are detected, which can be assigned to overtones and combination bands of the Te−Te stretching vibrations. However, the shoulder at 216 cm^−1^ is not present in this spectrum. The shift of the signal and the presence of overtones and combination bands indicate a resonance Raman effect. As the UV‐Vis spectra showed, the sample absorbs at 530 nm, which is close to the laser wavelength used. It seems plausible that the resonance Raman effect links the absorption and Raman scattering, proving that both are from the same species. The two Raman bands and the presence of combination bands suggest that this species is a higher telluride such as (Te_4_)^2−^ or (Te_5_)^2−^.

As with the UV‐Vis spectroscopy, we also analyzed a sample of K_2_Te_3_ dissolved in [P_66614_][OAc] as a reference. In the aqueous reaction medium, from which K_2_Te_3_ was crystalized, Albrecht et al. observed a single Raman signal at 181 cm ^− 1^ for (Te_2_)^2−^, measured with a 532 nm laser.^[31]^ After dissolution of K_2_Te_3_ in [P_66614_][OAc] the Raman spectrum shows two signals at 186 cm^−1^ and 156 cm^−1^ under 532 nm excitation and at 191 cm^−1^ and 156 cm^−1^ measured with 1064 nm laser. In contrast to the solution of tellurium in [P_66614_][OAc], the intensity of the band at 191 cm^−1^ is lower than that of the band at 156 cm^−1^, which is due to different ratio of the dissolved tellurides. When the 532 nm laser was used for excitation, the intensity at 186 cm^−1^ was higher than that at 156 cm^−1^. Again, the resonance Raman effect enhanced the signal from (Te_4_)^2−^ or (Te_5_)^2−^. We also observed overtones and combination bands with this laser (Figure S8). In summary, the Raman spectra confirm the dynamic equilibrium between different tellurides (Te_n_)^2−^ in the IL.

### Calculations on the tellurium cation (Te_4_)^2+^ and anions (Te_2_)^2−^, (Te_3_)^2−^, (Te_4_)^2−^ and (Te_5_)^2−^ with [P_4444_]^+^ or [OAc]^−^ counter ions

To get a better understanding of the tellurium species behind the Raman signals, we carried out DFT‐based calculations in a continuum solvent model for the clusters containing the cation (Te_4_)^2+^ and the anions (Te_2_)^2−^, (Te_3_)^2−^, (Te_4_)^2−^ and (Te_5_)^2−^ separately. As counterions, we used [OAc]^−^ for (Te_4_)^2+^, and [P_4444_]^+^ for the tellurides. We used [P_4444_]^+^ instead of [P_66614_]^+^ in the calculations to rationalize the computational demand by reducing the size and conformational space of the alkyl groups on the cation. Since the Raman spectra for solutions of tellurium in the two ILs were quite similar (Figure S8), we do not expect the alkyl chain length to have a decisive effect on the results.

**Figure 4:**
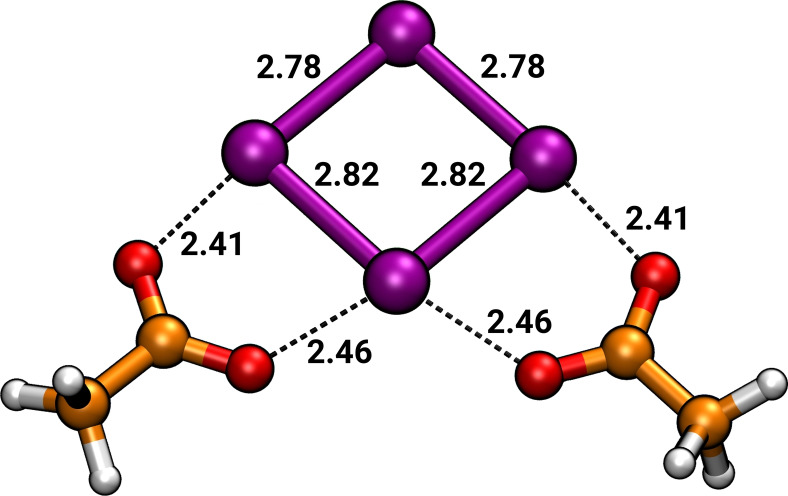
Te_4_[OAc]_2_ from geometry optimization. Short Te−O distances show a strong interaction of the oxygen atoms of the bidentate coordinating acetate to tellurium. All distances are in Å.

First, the cluster of the (Te_4_)^2+^ polycation with [OAc]^−^ counter ions is discussed. Upon optimization, the polycation adopts the well‐known planar square‐shaped geometry (Figure 4). Due to the delocalized charge distribution of the polycation, the anions do not coordinate a single tellurium atom, but bridge adjacent edges. The Te−O interaction is strong, as shown by the short bond distances in the optimized geometry, and the Mayer bond orders for these interactions (0.335, 0.333, 0.296, 0.293). To see whether the continuum model describes the solvation of this species in the IL, we performed ab initio molecular dynamics simulations of Te_4_[OAc]_2_ in [P_4444_][OAc] with periodic boundary conditions. The obtained Te−O distances show an excellent match with those in the static DFT calculations (cf. Figure 4 and Figure S15).

The calculated IR and Raman spectra are shown in Figure S9. The IR spectrum is dominated by a combined carboxylate bending and a C−C stretching of the acetate around 1368 cm^−1^. Additionally a strong IR signal is observed around 214 cm^−1^, which belongs to a Te−O stretching. The modes observed at 643 cm^−1^ and 926 cm^−1^ belong to bending motions of the carboxylate group. Increased Raman intensity of the Te_4_[OAc]_2_ complex is observed around 3020 cm^−1^, being related to C−H stretching motions of the methyl group attached to the acetate. Vibrational modes of the (Te_4_)^2+^ cation are observed around 175 cm^−1^ in the Raman spectrum, which is in good agreement with the vibrational frequencies in the power spectrum of the ab initio molecular dynamics trajectory (Figure S16). This Raman band is different from the one that was observed for (Te_4_)^2+^ were it was observed at 217 cm^−1^. However, this shift is probably due to the effect of the two coordinating acetate anions on the vibrations of the (Te_4_)^2+^. In the measured spectrum of dissolved elemental tellurium in the IL, no signal was observed around 175 cm^−1^. However, the nearby signals at 192 and 157 cm^−1^ may overlap the shifted signal of (Te_4_)^2+^.

**Figure 5:**
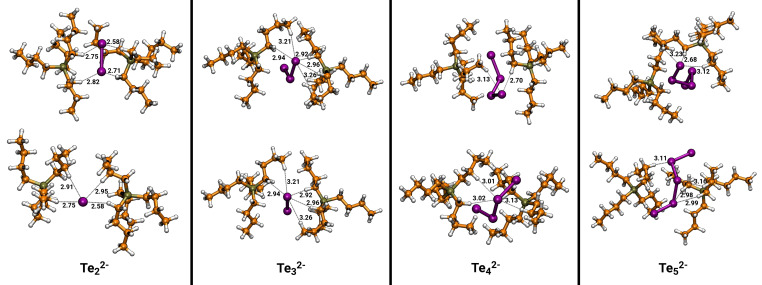
Calculated [P_4444_]_2_Te_x_ clusters in the gas phase. Interatomic Te−H distances are given in Å. The distance of the more acidic hydrogen atoms of the methylene group attached to the phosphorus lie mostly in the range of 2.6 to 3.0 Å were the less acidic hydrogen atoms show distances most slightly above 3.0 Å.

Next, clusters of the different tellurium polyanions (Te_n_)^2−^ (n=2–5) with the cation [P_4444_]^+^ of the IL are discussed. Figure 5 displays the resulting four clusters from two perspectives, respectively. Despite the different size of the anions, all clusters show remarkable similarities. Although the terminal tellurium atoms of the polyanions carry the majority of the negative charge, they do not interact directly with the phosphorus atom that formally carries the positive charge of the cation. Instead, solvation of the polyanions takes effect mostly through hydrogen atoms of the methylene groups attached to the phosphorus. These hydrogen atoms are known to be slightly acidic, as basic phosphonium ILs have shown the chemistry of phosphorus ylides.[^49^, ^50^, ^51^, ^52^] The intermolecular distances between these hydrogen atoms and the terminal tellurium atoms lie mostly between 2.6 Å and 3.0 Å. The distances between the tellurium atoms and some, less acidic, hydrogen atoms farther away from the phosphorus atom are somewhat longer, mostly slightly above 3.0 Å.

The corresponding IR and Raman spectra are shown in Figures S10 and S11, where Figure S10 shows the whole wavenumber region between 0 and 4000 cm^−1^ while Figure S11 only displays a magnification between 0 and 1700 cm^−1^. Both, the IR as well as the Raman spectra are dominated by high intensity bands within the C−H stretching region around 3000 cm^−1^. However, in contrast to the remaining clusters, [P_4444_]_2_Te_2_ shows additional bands around 2700 cm^−1^. These bands are also related to C−H stretching motions; however, they exclusively belong to hydrogen atoms attached to a methylene group next to the phosphorus atom and show a direct interaction with a tellurium atom. For the three clusters including the larger polyanions, the C−H stretching of these particular hydrogen atoms is located around 3000 cm^−1^. In the experimental spectra, bands around 2700 cm^−1^ are always present, also in the IL without dissolved tellurium, indicating that the [OAc]^−^ anion also interacts with the methylene groups next to the phosphorus atom. Figure S11 reveals further differences (especially for the Raman spectra) between the cluster [P_4444_]_2_(Te_2_) with the smallest polyanion and the remaining clusters. While the clusters containing the (Te_3_)^2−^, (Te_4_)^2−^ and (Te_5_)^2−^ polyanion show similar Raman spectra considering peak locations and intensities, the cluster [P_4444_]_2_Te_2_ shows increased Raman intensity between 500 cm^−1^ and 1500 cm^−1^. Within this cluster, Raman signals can be assigned to the following modes: 573 cm^−1^: C−P stretching; 591 cm^−1^ and 681 cm^−1^: C−P stretching combined with a twisting of methylene groups that feature a hydrogen bond to a tellurium atom; 750 cm^−1^, 807 cm^−1^ and 814 cm^−1^: twisting of methylene groups; 891 cm^−1^ and 902 cm^−1^: scissoring of methylene groups; 1106 cm^−1^: combined twisting and scissoring of methylene groups; 1376 cm^−1^: wagging of methylene groups; 1400 cm^−1^, 1433 cm^−1^ and 1500 cm^−1^: scissoring of methylene groups.

Within the remaining clusters the assignment of modes is similar as for the [P_4444_]_2_Te_2_, however, only [P_4444_]_2_Te_2_ shows increased Raman activity for modes that include hydrogen atoms bound to a tellurium atom. Most likely this is due to the fact that (Te_2_)^2−^ has a higher charge density than the longer (Te_n_)^2−^ chains and thus, forms stronger hydrogen bonds to the phosphonium cation. The vibrations of the tellurium polyanions show only very weak IR and Raman activity. The Te−Te stretching within (Te_2_)^2−^ is located around 162 cm^−1^, for (Te_3_)^2−^ the symmetric and asymmetric stretching are located at 165 cm^−1^ and 167 cm^−1^, respectively. In the experimental spectra both stretching modes are hardly distinguishable from each other and appear as one signal. For (Te_4_)^2−^ the Te−Te stretching of the central bond is located at 132 cm^−1^ while the stretching motions including the terminal tellurium atoms are located at 162 cm^−1^ and 172 cm^−1^. Indeed the mode located at 172 cm^−1^ shows increased IR intensity and Raman activity, yet it is a combination of the Te−Te stretching and a twisting of several methylene groups. The (Te_5_)^2−^ polyanion shows a very weak Te−Te stretching around 130 cm^−1^ and 145 cm^−1^, however, the most intense vibrations are located at 175 cm^−1^ and 183 cm^−1^ involving combined stretching motions of all tellurium atoms. The results of the quantum chemical calculations support our hypothesis that long chain tellurides (n>3) in IL solution are traceable by additional signals above 180 cm^−1^ in the Raman spectrum while (Te_2_)^2−^ and (Te_3_)^2−^ show signals around 160 cm^−1^.

### Cyclic voltammetry and electrodeposition: Proof of tellurium cations and anions in solution

UV‐Vis and Raman spectroscopy demonstrated the formation of tellurides (Te_n_)^2−^ when tellurium is dissolved in [P_66614_][OAc] beyond doubt, although the evidence for tellurium cations was weak. Cyclic voltammetry (CV) allows for studying cations and anions in one cycle without changing the experimental setup. However, the composition of the solution can change by the precipitation of elemental tellurium under the influence of air. To avoid this problem, an inert measurement setup was developed. An electrochemical cell with two additional openings was provided with two septa through which a controlled, weak argon stream was flushed through the cell via cannulas. The slight argon overpressure largely prevented contamination with air. With this setup, the solution generally retained its color for more than four hours. Only the CV cycles themselves affected the composition of the solution. The measurement program included a potential range of −3.5 V to 3 V.

**Figure 6:**
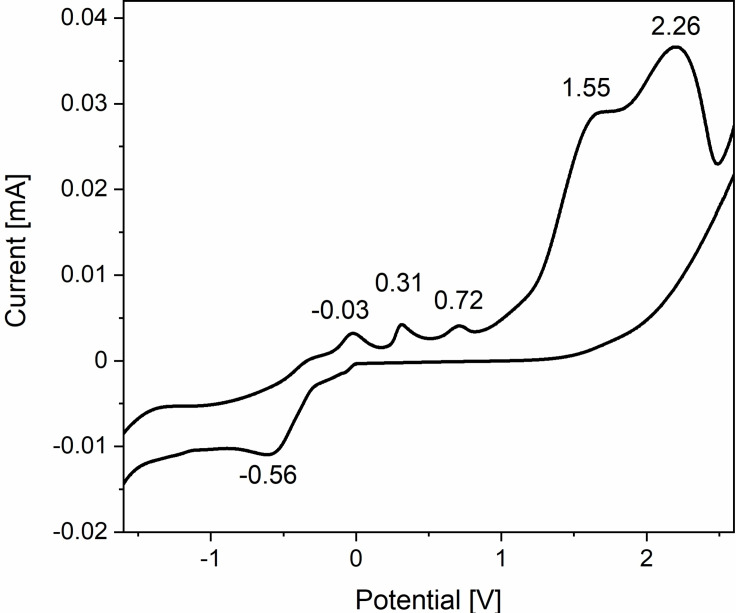
Section of the cyclic voltammogram (full range −3.5 V to 3.0 V) of a solution of tellurium in [P_66614_][OAc] measured at 100 °C in an argon flushed cell.

At the scan rate of 30 mV s^−1^, the slow movement of ions in the IL, which is still quite viscous at 100 °C, combined with the low tellurium concentration prevented the detection of peaks in the CV. In contrast, the scan rate of 5 mV s^−1^ proved to be suitable. When the measurement started with a negative potential (reduction), a clear reduction signal appeared at a potential of −0.56 V (Figure 6). As the potential is subsequently increased, five oxidation signals were observed, weak ones at −0.03 V, 0.31 V, 0.71 V, and stronger ones at 1.55 V and 2.26 V. The second cycle showed three reduction signals: very weak ones at −0.34 V and −0.89 V and a step at about −1.48 V, indicating a change in the system in the first cycle.

When the measurement started with a positive potential (oxidation), the first signal at −0.03 V was not observed and the other oxidation signals shift to 0.12 V, 0.81 V, 1.78 V and 2.53 V (Figure S12). Reduction signals were found at −0.32 V, −1.24 V, and −0.87 (shoulder). In the second cycle, which began with the reduction, the signal at about 0 V appeared again.

After the CV measurements, the supernatant was collected under inert conditions and Raman (Figure S13) and UV‐Vis spectra (Figure S14) were recorded. The intensity of the signals assigned to tellurides had decreased and almost disappeared.

The CVs show that redox‐active anions and cations are already present in the solution at the beginning of the measurements. Since the IL is electrochemically inert in this potential window, these can only be tellurium ions. The redox chemistry is complex and is altered by the partial deposition of tellurium that occurs during CV. Various tellurium anions are present in the original solution of tellurium in [P_66614_][OAc], but only one cation dominates.

**Figure 7:**
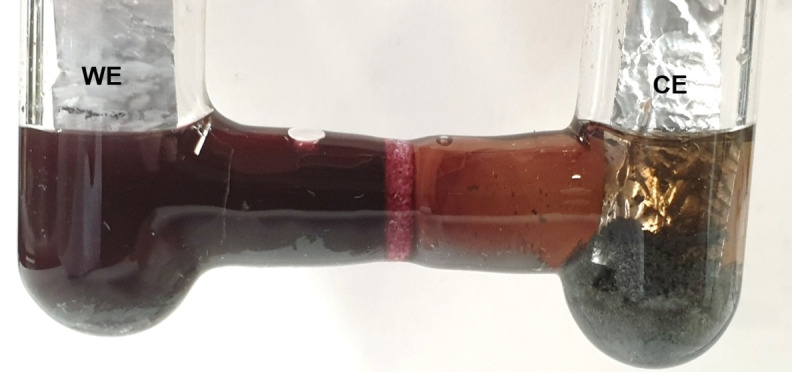
H‐tube used for electrodeposition under inert conditions. A potential of −3.7 V was applied for 16 h to a solution of tellurium in [P_66614_][OAc] at 100 °C. The current was about 0.4 mV. A greyish powder of tellurium formed in both electrode compartments. The solution at the working electrode (WE) is still intensely colored purple, while the solution at the counter electrode (CE) has turned translucent brown.

For the electrolytic deposition of tellurium, an H‐tube was used, equipped with two additional valves for the argon supply to meet the requirements for an inert atmosphere. Separation of the electrode compartments was established by a glass filter so that both sides of the H‐tube were connected only by the liquid phase. The tube was filled with the red solution of tellurium dissolved in [P_66614_][OAc] (Figure 7). Strips of polished aluminum foil, washed with deionized water and acetone, served as electrodes in a two‐electrode setup. The cell potential was gradually increased from −2 V to −3.7 V and held at this potential. A current of about 0.4 mV occurred. To decrease the viscosity of the solution, the H‐tube was heated to 100 °C in an oil bath during the experiment.

**Figure 8:**
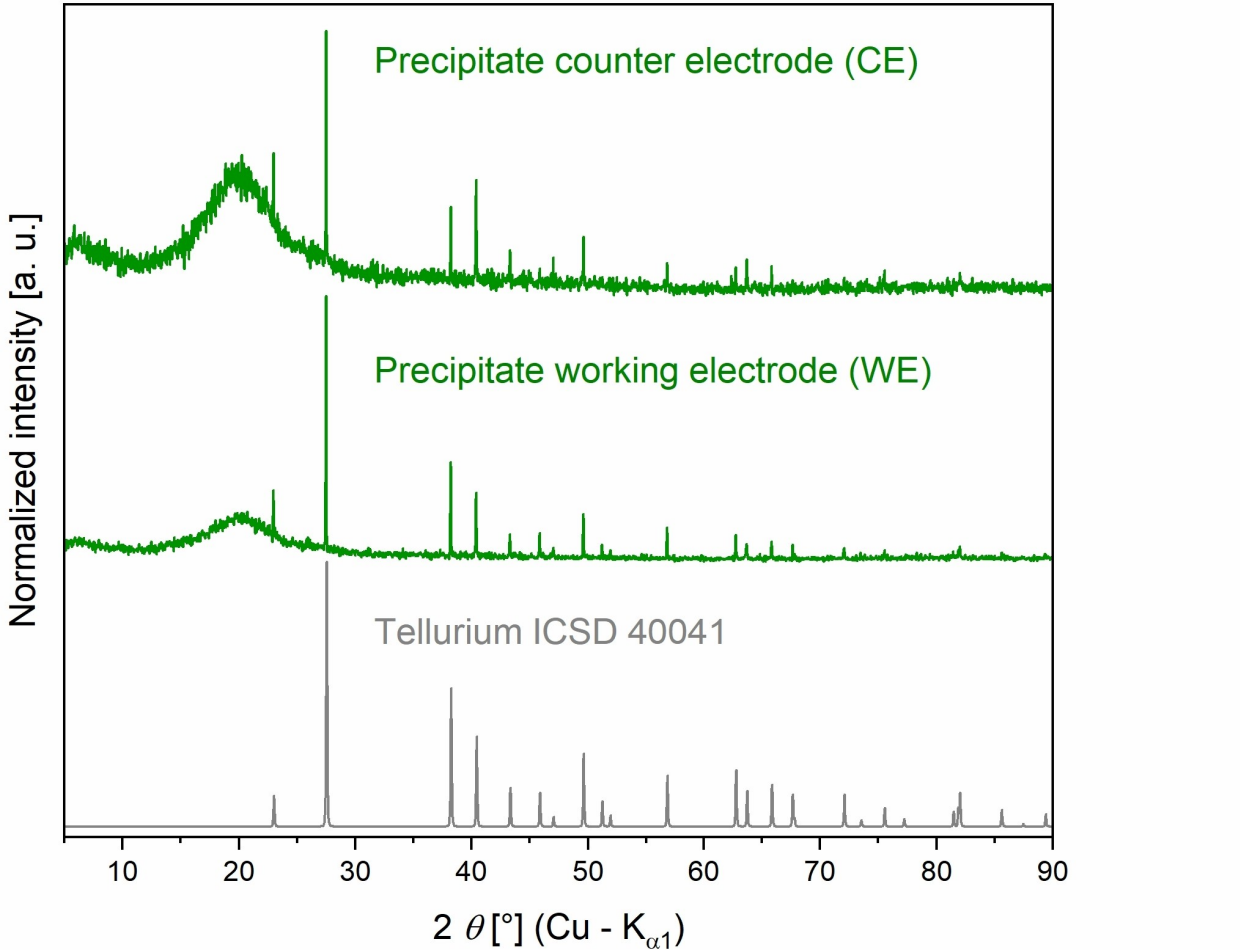
PXRD of the precipitate in the electrode spaces of working electrode (WE) and counter electrode (CE). Both show only reflections for tellurium in comparison with simulated PXRD pattern, simulated from single crystal data from the inorganic structure database (ICSD), which proves that tellurium is coexisting as cations and anions when dissolved in [P_66614_][OAc].

After 16 h, the solution around the counter electrode (CE) had almost completely decolorized and there was a gray powder below both electrodes (Figure 7). The solution at the working electrode (WE) still had a deep red color. After separately removing the IL from each electrode compartment under argon atmosphere, the electrodes still appeared intact, with only a slightly darkened surface at the WE. UV‐Vis spectra of the supernatants from the CE compartment showed a strong decrease of the absorbance by tellurides at 533 nm and 376 nm, in contrast to the supernatant on the WE side (Figure S14). The precipitated powders were identified as pure grey tellurium by PXRD in both cases (Figure 8). No trace of aluminum telluride was found. Electrodeposition of tellurium at both electrodes vividly confirmed that tellurium disproportionate at 100 °C in [P_66614_][OAc].

## Conclusion

Gray tellurium dissolves in [P_66614_][OAc] at 50 °C to form a dark red solution, but the process proceeds more rapidly at 100 °C. The IL is not chemically affected in this process; only trace impurities of alkyl phosphanes react with tellurium to form phosphane tellurides. Purification of the IL is possible, but is not necessary for the given purpose. Extensive spectroscopic and electrochemical studies showed that tellurium disproportionate in the IL. In this reaction, tellurium anions (Te_*n*_)^2−^ with chain lengths up to at least *n*=5 form, which are in dynamic equilibrium with each other. Incidentally, the dynamic breaking and reformation of Te−Te bonds at temperatures as low as 50 °C explains why there is no crystalline tellurium allotrope with ring‐shaped molecules. The cation side is dominated by a species that we have not been able to identify with certainty, but which is very likely the aromatic four‐membered ring (Te_4_)^2+^. DFT calculations indicate that the phosphonium IL does not coordinate the tellurium ions, but merely stabilizes them via electrostatic interactions. The latter seem to be sufficient to initiate the dissolution process of tellurium already near room temperature. This gives us a deeper understanding in the dissolution process in ionic liquids for a more rational synthetic design in materials chemistry in ILs.

## Experimental Section

**General remarks**: The starting materials and the products were handled in an argon‐filled glove‐box (MBraun UNILab) with *p*(O_2_)/*p* ^0^ <1 ppm and *p*(H_2_O)/*p* ^0^ <1 ppm. Tellurium (Chempur, 99.9999 %, bar) was reduced in a hydrogen flow at 400 °C. DCM (Fisher Scientific, HPLC grade, amylene stabilized), EtOH (Fisher Scientific, absolute 99.8 %, Certified AR for Analysis) and THF (VWR Chemicals, HPLC grade) were degassed and dried in an MBraun SPS. Enriched ^125^Te (99.7 %) for NMR experiments was purchased from Coretechnet. [P_66614_]Cl >95 % and [P_4444_]Cl >95 % was purchased from IoLiTec. [P_66614_]Cl was either used directly for the synthesis of [P_66614_][OAc] (see below) or purified in advance (see below).

**Ionic liquid synthesis**: Both phosphonium acetate ILs were synthesized by anion metathesis as previously published with slight modifications.^[15]^ Either [P_4444_]Cl (23,6 g, 80 mmol) or [P_66614_]Cl (41.6 g, 80 mmol) was dissolved in 40 mL EtOH, combined under vigorous stirring with 100 mL ethanolic solution of 4.9 g (excess, 50 mmol) potassium acetate and stirred at room temperature for 16 h. Precipitated potassium chloride was filtered off. After removal of the solvent on a rotary evaporator, during which more salt precipitated, the IL was filtered again, dried under vacuum at 110 °C ([P_66614_][OAc]) or 80 °C ([P_4444_][OAc]) and 10^−3^ mbar and transferred for storage into the glove box. The melting point of 58±0.1 °C was determined for [P_4444_][OAc] with a Electrothermal IA9100 digital melting point apparatus with a heating rate of 1 K/min. The glass tube used for the measurement were field and sealed inside the glovebox.

**Purification of [P_66614_]Cl and [P_66614_][OAc]**: Either the commercially purchased [P_66614_]Cl was purified and afterwards used for the synthesis of [P_66614_][OAc] or the unpurified [P_66614_]Cl was used for the synthesis and the product was purified. A higher yield was achieved if [P_66614_]Cl was purified before the synthesis of [P_66614_][OAc]. In both cases almost the same purification protocol was used except for the first purification step, the neutralization with 0.1 M NaOH solution, which was not used for purification of [P_66614_][OAc] after the synthesis. The purification protocol was already published in a previous publication.^[24]^

**Sample preparation**: In a usual sample preparation, 20 mg of elemental tellurium was weighed in a 5 ml glass flask with a magnetic stirring bar. The tellurium was then covered with 1 g of purified or unpurified [P_66614_][OAc]. The chemicals were handled in the glove box under argon atmosphere. The Flask was sealed with a glass plug and vacuum grease, removed from the glove box, and heated in an oil bath to 60 °C for 18 h. After this treatment, the flask was transferred into the glove box again and IL‐solution was filtered with syringe filter (pore width 0.45 μm) to remove undissolved tellurium.

**Electrochemical measurements**: In a 25 mL round bottom flask equipped with a magnetic stirring bar, 300 mg freshly ground Te (2.35 mmol) was placed and covered with 20 g of [P_66614_][OAc] and sealed with a glass stop with silicon grease. After heating for 16 h at 100 °C, in an oil‐bath outside the glovebox the flask was brought back into a glovebox and the solution filtrated with a syringe filter to remove rests of undissolved Te. All electrochemical experiments were performed on a VMP‐3 potentiostate form BioLogic controlled by EC‐LAB Electrochemistry software. The cyclic voltammogram (CV) was recorded using a three‐electrode setup (Wuhan Corrtest Instruments Corp. Ltd.). The electrodeposition experiments were performed in potential control method, using two aluminum stripes as electrodes in an H‐type glassware.

**Cyclic voltammetry measurements**: For the measurements, 4 mL of the solution were placed inside a glovebox in the glass container of the electrochemical cell. The cyclic voltammogram (CV) was recorded using a three‐electrode setup (Wuhan Corrtest Instruments Corp. Ltd.) with a glassy carbon disk working electrode (diameter 3 mm), a platinum wire (Pt, 99.95 %, 0.5 mm diameter) as counter electrode and a platinum plate (99.95 %, 10×10×0.1 mm) as pseudo‐reference electrode in the range of −3.5 V to 3 V and with a scan rate of 5 mV s^−1^. Prior to the measurement, the platinum electrodes were rinsed with acetone and cleaned with fuzz‐free tissue, before drying on air and moving inside the glovebox. While the measurement the cell was placed in an oil‐bath and heated to 100 °C. The cell was mounted with two septa on the top of the cap and flushed with argon via two 0.8 mm cannulas. Working with an only under argon closed cell leads to precipitation of Te even bevor the measurement starts, due to entering of air.

**Electrodeposition**: For electrodeposition of Te from the solutions, a H‐shaped glassware was used. The two sides for the electrode in this glassware were separated by a glass sieve and have two valves on top for gas supply. Two aluminum foil stripes witch were grinded with BUEHLER Carbimet Grit 1000 [P2500] served as electrodes. After grinding, the surfaces of these strips were rinsed with deionized water and acetone and then air‐dried. The H‐shaped glassware was equipped with the electrodes inside a glovebox and filled with approximately 10 mL of solution from both sides, until the connecting bridge was filled completely. Afterwards the glassware was sealed with two septa and positioned upright in an oil‐bath outside the glovebox, which was tempered to 100 °C. Inert conditions inside the glassware were ensured by a constant argon stream during the experiment. The electrodes were connected to the electronic measurement system and cell potential was gradually increased from −2 V to −3.7 V and held at this potential for 16 h. A current of about 0.4 mV occurred.

**Nuclear magnetic resonance spectroscopy**: All samples for liquid state NMR measurements were prepared in the glove box under argon atmosphere and in low pressure/vacuum 5 mm NMR tubes sealed with polytetrafluoroethylene caps from Deutero. Capillaries filled with DMSO‐d6 were added to the IL sample inside the NMR tube to ensure field‐frequency lock. The NMR spectra were recorded with a Bruker Avance Neo 300 MHz spectrometer with a 5 mm high‐resolution probe. For ^31^P NMR spectra a transmitter frequency of 121.49 MHz, a relaxation delay of 10 s and a pulse length of 8.3 μs were used to record 128 scans. The chemical shifts in the ^31^P NMR spectra were referenced relative to H_3_PO_4_. The ^125^Te NMR spectra were recorded at the transmitter frequency 94.65 MHz using 16000 to 32000 scans. In order to shorten the relaxation time and increase the excitation bandwidths, a 30° instead of a 90° pulse with a pulse length of 3.33 μs and relaxation delay of 5 s was used. To scan a large chemical shift range from 7000 to −4000 ppm the transmitter frequency offset was varied in steps of 104159.35 Hz. The chemical shift was measured relative to Me_2_Te (δ=0 ppm). For calibration the secondary external standard Te(OH)_6_ (δ=707 ppm) was used.^[53]^

**Raman spectroscopy**: The Raman spectra were recorded with a Bruker RFS 100 Fourier transform Raman spectrometer with a 1064 nm Nd‐YAG‐Laser and a Thermo Fischer DXR SmartRaman spectrometer with a 532 nm frequency doubled Nd‐YAG‐Laser. For the 1064 nm excitation, we used a laser power of 426 mW and recorded 500 scans. For the 532 nm excitation we used a laser power of 8 mW and 100 scans. With a 532 nm excitation all spectra showed fluorescence which was corrected by a 5^th^ order polynomial fluorescence correction. Air sensitive samples were prepare in the glove box under argon atmosphere in capillary tubes and sealed with modelling clay.

**UV‐Vis spectroscopy**: The Absorption spectra were recorded with a VARIAN CARY 50 UV/Vis‐ spectrometer. The samples were prepared either in 0.1 cm quartz cuvettes or as thin layer between two 1 cm×1 cm sapphire glass plates. All samples were prepared in the glove box under argon atmosphere, transported to the spectrometer in an air‐sealed container and measured immediately. The cuvettes were covered with foil (PARAFILM) in addition.

**Powder X‐ray diffraction (PXRD)**: X‐ray diffraction data of precipitated powders were collected at 296(1) K on an X′Pert Pro MPD diffractometer (PANalytical), equipped with a curved Ge(111) monochromator, using Cu‐K*α*_1_ radiation (*λ*=154.056 pm) and identified via comparison with simulated diffraction patterns of single crystal tellurium, from single crystal data of the inorganic crystal structure database (ICSD No. 40041). Simulation of the patterns was done via the software STOE WinXPOW version 2.08.

**Quantum chemical calculations**: The structures of the clusters of tellurium cations and anions with [P_4444_][OAc] have been generated by running molecular dynamics (MD) simulations of the clusters as implemented in the xtb program package.^[54]^ From all MD steps, the lowest energy structure has been taken as input for all following quantum chemical calculations which have been performed with the Orca program package.^[55]^ As level of theory for the quantum chemical calculations, B97‐3c^[56]^ (internally employing a reasonably large modified TZVP AO basis set) has been employed for geometry optimizations as well as frequency calculations. Tight convergence criteria (TightSCF and TightOpt) have been employed for the SCF calculations as well as for the geometry optimizations. As DFT integration grid, grid4 and grid5 have been chosen for the geometry optimizations and the frequency calculations, respectively. The polarizability for obtaining the Raman spectra has been calculated analytically.

**Ab initio molecular dynamics simulations**: For the ab initio molecular dynamics (AIMD) simulation of Te_4_[OAc]_2_ in [P_4444_][OAc], a simulation box containing 30 ion pairs of the IL and one unit of Te_4_[OAc]_2_ were set up using Packmol.[^57^, ^58^] The edge length of the cubic simulation box was 2694.44 pm. The simulations were carried out based on the hybrid Gaussian and plane wave (GPW) method using the CP2 K program package^[59]^ and the Quickstep^[60]^ module. As level of theory the BLYP functional,[^61^, ^62^] the corresponding BLYP Goedecker‐Tetter‐Hutter pseudopotentials[^63^, ^64^, ^65^] and the molecularly optimized double‐zeta basis set (MOLOPT‐DZVP‐SR‐GTH)^[66]^ were employed. Dispersion interactions were taken care of by the D3 dispersion correction scheme.[^67^, ^68^] As ensemble, the NVT ensemble has been employed and the timestep has been set to 0.5 fs. The simulations consist of an initial geometry optimization on order to remove energy hotspots possibly arising from the packing procedure. Afterwards, an equilibration run followed for 10000 timesteps at a temperature of 600 K. For the following production run, the temperature was decreased to 420 K and a trajectory of 40000 timesteps has been produced. The trajectory obtained from the production run has been used for further analysis using TRAVIS.[^69^, ^70^]

## Conflict of interest

The authors declare no conflict of interest.

1

## Supporting information

As a service to our authors and readers, this journal provides supporting information supplied by the authors. Such materials are peer reviewed and may be re‐organized for online delivery, but are not copy‐edited or typeset. Technical support issues arising from supporting information (other than missing files) should be addressed to the authors.

Supporting Information

## Data Availability

The data that support the findings of this study are available in the supplementary material of this article.

## References

[1] A. J. Greer , J. Jacquemin , C. Hardacre , Molecules 2020, 25, 5207. 10.3390/molecules25215207 PMC7664896 33182328

[2] J. D. Holbrey , K. R. Seddon , Clean Technol. Environ. Policy 1999, 1, 223–236. 10.1007/s10098-020-01965-1 PMC7581307 33110403

[3] P. Wasserscheid , W. Keim , Angew. Chem. Int. Ed. 2000, 39, 3772–3789; 10.1002/1521-3773(20001103)39:21<3772::aid-anie3772>3.0.co;2-5 11091453

[4] A. Taubert , Z. Li , Dalton Trans. 2007, 723–727. 17279241 10.1039/b616593a

[5] Z. Ma , J. Yu , S. Dai , Adv. Mater. 2010, 22, 261–285. 20217687 10.1002/adma.200900603

[6] D. Freudenmann , S. Wolf , M. Wolff , C. Feldmann , Angew. Chem. Int. Ed. 2011, 50, 11050–11060; 10.1002/anie.201100904 21990270

[7] G. G. Eshetu , M. Armand , B. Scrosati , S. Passerini , Angew. Chem. Int. Ed. 2014, 53, 13342–13359; 10.1002/anie.201405910 25303401

[8] T. Zhang , T. Doert , H. Wang , S. Zhang , M. Ruck , Angew. Chem. Int. Ed. 2021, 60, 22148–22165; 10.1002/anie.202104035 PMC8518931 34032351

[9] H. Liu , Y. Liu , J. Li , Phys. Chem. Chem. Phys. 2010, 12, 1685–1697. 20145833 10.1039/b921469k

[10] K. J. Fraser , D. R. MacFarlane , Aust. J. Chem. 2009, 62, 309–321.

[11] A. B. Pereiro , J. M. M. Araújo , F. S. Oliveira , J. M. S. S. Esperança , J. N. Canongia Lopes , I. M. Marrucho , L. P. N. Rebelo , J. Chem. Thermodyn. 2012, 55, 29–36.

[12] K. Oster , P. Goodrich , J. Jacquemin , C. Hardacre , A. P. C. Ribeiro , A. Elsinawi , J. Chem. Thermodyn. 2018, 121, 97–111.

[13] B. Wang , L. Qin , T. Mu , Z. Xue , G. Gao , Chem. Rev. 2017, 117, 7113–7131. 28240867 10.1021/acs.chemrev.6b00594

[14] S. Chowdhury , R. S. Mohan , J. L. Scott , Tetrahedron 2007, 63, 2363–2389.

[15] A. J. Holding , M. Heikkilä , I. Kilpeläinen , A. W. T. King , ChemSusChem 2014, 7, 1422–1434. 24616349 10.1002/cssc.201301261

[16] D. R. del Cerro , T. V. Koso , T. Kakko , A. W. T. King , I. Kilpeläinen , Cellulose 2020, 27, 5545–5562.

[17] W. Shi , R. L. Thompson , E. Albenze , J. A. Steckel , H. B. Nulwala , D. R. Luebke , J. Phys. Chem. B 2014, 118, 7383–7394. 24927032 10.1021/jp502425a

[18] C. A. Pena , A. Soto , H. Rodríguez , Chem. Eng. J. 2021, 409, 128191.

[19] K. Oster , C. Hardacre , J. Jacquemin , A. P. C. Ribeiro , A. Elsinawi , Pure Appl. Chem. 2019, 91, 1309–1340.

[20] È. Boros , M. J. Earle , M. A. Gîlea , A. Metlen , A. V. Mudring , F. Rieger , A. J. Robertson , K. R. Seddon , A. A. Tomaszowska , L. Trusov , J. S. Vyle , Chem. Commun. 2010, 46, 716–718. 10.1039/b910469k 20087497

[21] A. Wolff, Dissertation, TU Dresden (Germany), **2018**.

[22] T. Zhang , T. Doert , K. Schwedtmann , J. J. Weigand , M. Ruck , Dalton Trans. 2020, 49, 1891–1896. 31967632 10.1039/c9dt04604f

[23] T. Zhang, Dissertation, TU Dresden (Germany), **2018**.

[24] M. A. Grasser , T. Pietsch , E. Brunner , T. Doert , M. Ruck , ChemistryOpen 2021, 10, 117–124. 33565727 10.1002/open.202000249 PMC7874243

[25] J. L. McAfee , J. R. Andreatta , R. S. Sevcik , L. D. Schultz , J. Mol. Struct. 2012, 1022, 68–71.

[26] W. Klemm , H. Sodomann , P. Langmesser , Z. Anorg. Allg. Chem. 1939, 241, 281–304.

[27] L. D. Schultz , W. H. Koehler , Inorg. Chem. 1987, 26, 1989–1993.

[28] L. D. Schultz , Inorg. Chim. Acta 1990, 176, 271–275.

[29] M. Björgvinsson , G. J. Schrobilgen , Inorg. Chem. 1991, 30, 2540–2547.

[30] R. G. Teller , L. J. Krause , R. C. Haushalter , Inorg. Chem. 1983, 22, 1809–1812.

[31] R. Albrecht , M. Ruck , Angew. Chem. Int. Ed. 2021, 60, 22570–22577; 10.1002/anie.202107642 PMC8518872 34375499

[32] M. F. Groh , A. Wolff , M. A. Grasser , M. Ruck , Int. J. Mol. Sci. 2016, 17, 1452. 10.3390/ijms17091452 PMC5037731 27598123

[33] E. Ahmed , E. Ahrens , M. Heise , M. Ruck , Z. Anorg. Allg. Chem. 2010, 636, 2602–2606.

[34] E. Ahmed , J. Beck , J. Daniels , T. Doert , S. J. Eck , A. Heerwig , A. Isaeva , S. Lidin , M. Ruck , W. Schnelle , A. Stankowski , Angew. Chem. Int. Ed. 2012, 51, 8106–8109; 10.1002/anie.201200895 22764118

[35] T. W. Couch , D. A. Lokken , J. D. Corbett , Inorg. Chem. 1972, 11, 357–362.

[36] D. Freudenmann , C. Feldmann , Z. Anorg. Allg. Chem. 2011, 637, 1481–1485.

[37] C. Schulz , J. Daniels , T. Bredow , J. Beck , Angew. Chem. Int. Ed. 2016, 55, 1173–1177; 10.1002/anie.201507644 26632775

[38] X. Qu , G. Zhou , R. Wang , B. Yuan , M. Jiang , J. Tang , Green Chem. 2021, 23, 1871–1882.

[39] J. Barr , R. Gillespie , R. Kappoor , G. P. Pez , J. Am. Chem. Soc. 1968, 90, 6855–6856.

[40] R. J. Myers , J. Solution Chem. 2007, 36, 395–403.

[41] T. Zhang , K. Schwedtmann , J. J. Weigand , T. Doert , M. Ruck , Chem. Eur. J. 2018, 24, 9325–9332. 29762892 10.1002/chem.201800320

[42] A. Nordheider , J. D. Woollins , T. Chivers , Chem. Rev. 2015, 115, 10378–10406. 26312498 10.1021/acs.chemrev.5b00279

[43] G. J. Schrobilgen , R. C. Burns , P. Granger , J. Chem. Soc. Chem. Commun. 1978, 21, 957–960.

[44] R. C. Burns , R. J. Gillespie , W.-C. Luk , D. R. Slim , Inorg. Chem. 1979, 18, 3086–3094.

[45] R. C. Burns , R. J. Gillespie , Spectrochim. Acta Part A 1983, 39, 439–447.

[46] J. Beck , Coord. Chem. Rev. 1997, 163, 55–70.

[47] L. D. Schultz , E. T. Davis , J. D. Lewis , Spectrosc. Lett. 2013, 46, 191–194.

[48] F. Watari , Inorg. Chem. 1981, 20, 1776–1779.

[49] D. S. Firaha , A. V. Gibalova , O. Hollóczki , ACS Omega 2017, 2, 2901–2911. 31457625 10.1021/acsomega.7b00230 PMC6641186

[50] T. R. Gohndrone , T. Bum Lee , M. A. DeSilva , M. Quiroz-Guzman , W. F. Schneider , J. F. Brennecke , ChemSusChem 2014, 7, 1970–1975. 24801593 10.1002/cssc.201400009

[51] T. Bum Lee , S. Oh , T. R. Gohndrone , O. Morales-Collazo , S. Seo , J. F. Brennecke , W. F. Schneider , J. Phys. Chem. B 2016, 120, 1509–1517. 26556283 10.1021/acs.jpcb.5b06934

[52] A. C. Vetter , D. G. Gilheany , K. Nikitin , Org. Lett. 2021, 23, 1457–1462. 33529039 10.1021/acs.orglett.1c00133

[53] B. W. Tötsch , P. Peringer , F. Sladky , J. Chem. Soc. Chem. Commun. 1981, 841–842.

[54] C. Bannwarth , S. Ehlert , S. Grimme , J. Chem. Theory Comput. 2019, 15, 1652–1671. 30741547 10.1021/acs.jctc.8b01176

[55] F. Neese , WIREs Comput. Mol. Sci. 2012, 2, 73–78.

[56] J. G. Brandenburg , C. Bannwarth , A. Hansen , S. Grimme , J. Chem. Phys. 2018, 148, 064104. 29448802 10.1063/1.5012601

[57] L. Martínez , R. Andrade , E. G. Birgin , J. M. Martínez , J. Comput. Chem. 2009, 30, 2157–2164. 19229944 10.1002/jcc.21224

[58] J. M. Martínez , L. Martínez , J. Comput. Chem. 2003, 24, 819–825. 12692791 10.1002/jcc.10216

[59] T. D. Kühne , M. Iannuzzi , M. Del Ben , V. V. Rybkin , P. Seewald , F. Stein , T. Laino , R. Z. Khaliullin , O. Schütt , F. Schiffmann , D. Golze , J. Wilhelm , S. Chulkov , M. H. Bani-Hashemian , V. Weber , U. Borštnik , M. Taillefumier , A. S. Jakobovits , A. Lazzaro , H. Pabst , T. Müller , R. Schade , M. Guidon , S. Andermatt , N. Holmberg , G. K. Schenter , A. Hehn , A. Bussy , F. Belleflamme , G. Tabacchi , A. Glöß , M. Lass , I. Bethune , C. J. Mundy , C. Plessl , M. Watkins , J. VandeVondele , M. Krack , J. Hutter , J. Chem. Phys. 2020, 152, 194103. 33687235 10.1063/5.0007045

[60] J. VandeVondele , M. Krack , F. Mohamed , M. Parrinello , T. Chassaing , J. Hutter , Comput. Phys. Commun. 2005, 167, 103–128.

[61] J. P. Perdew , Phys. Rev. B 1986, 33, 8822–8824. 10.1103/physrevb.33.8822 9938299

[62] A. D. Becke , Phys. Rev. A 1988, 38, 3098–3100. 10.1103/physreva.38.3098 9900728

[63] S. Goedecker , M. Teter , J. Hutter , Phys. Rev. B 1996, 54, 1703–1710. 10.1103/physrevb.54.1703 9986014

[64] C. Hartwigsen , S. Goedecker , J. Hutter , Phys. Rev. B 1998, 58, 3641–3662.

[65] M. Krack , Theor. Chem. Acc. 2005, 114, 145–152.

[66] J. VandeVondele , J. Hutter , J. Chem. Phys. 2007, 127, 114105. 17887826 10.1063/1.2770708

[67] S. Grimme , J. Antony , S. Ehrlich , H. Krieg , J. Chem. Phys. 2010, 132, 154104. 20423165 10.1063/1.3382344

[68] S. Grimme , S. Ehrlich , L. Goerigk , J. Comput. Chem. 2011, 32, 1456–1465. 21370243 10.1002/jcc.21759

[69] M. Brehm , B. Kirchner , J. Chem. Inf. Model. 2011, 51, 2007–2023. 21761915 10.1021/ci200217w

[70] M. Brehm , M. Thomas , S. Gehrke , B. Kirchner , J. Chem. Phys. 2020, 152, 164105. 32357781 10.1063/5.0005078

