# Supplementary material for: Coexistence of Tellurium Cations and Anions in Phosphonium‐Based Ionic Liquids
Source: Chemistry. 2021 Dec 28;28(7):e202103770. doi: 10.1002/chem.202103770 (PMC9304316; doi:10.1002/chem.202103770)
Supplement: Supplementary file 1 — Supporting Information [file CHEM-28-0-s001.pdf]

Chemistry–A European Journal

Supporting Information

Coexistence of Tellurium Cations and Anions in Phosphonium-Based Ionic Liquids

Matthias A. Grasser, Tobias Pietsch, Jan Blasius, Oldamur Hollóczki, Eike Brunner, Thomas Doert, and Michael Ruck*

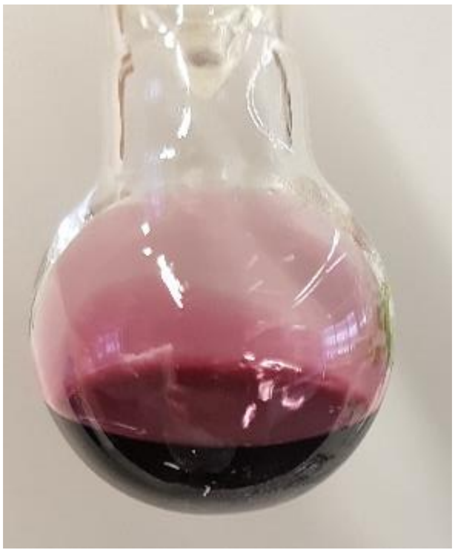

Figure S1. [P_{66614}][OAc] after the dissolution of tellurium.

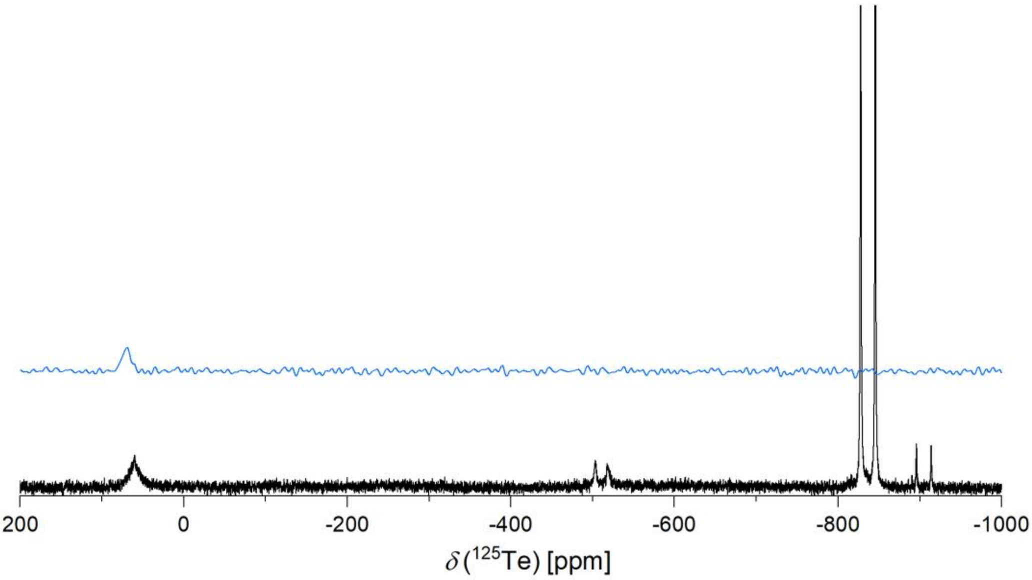

Figure S2. ^{125}Te-NMR of ^{125}Te enriched tellurium dissolved in [P_{66614}][OAc] (black) or [P_{4444}][OAc] (blue).

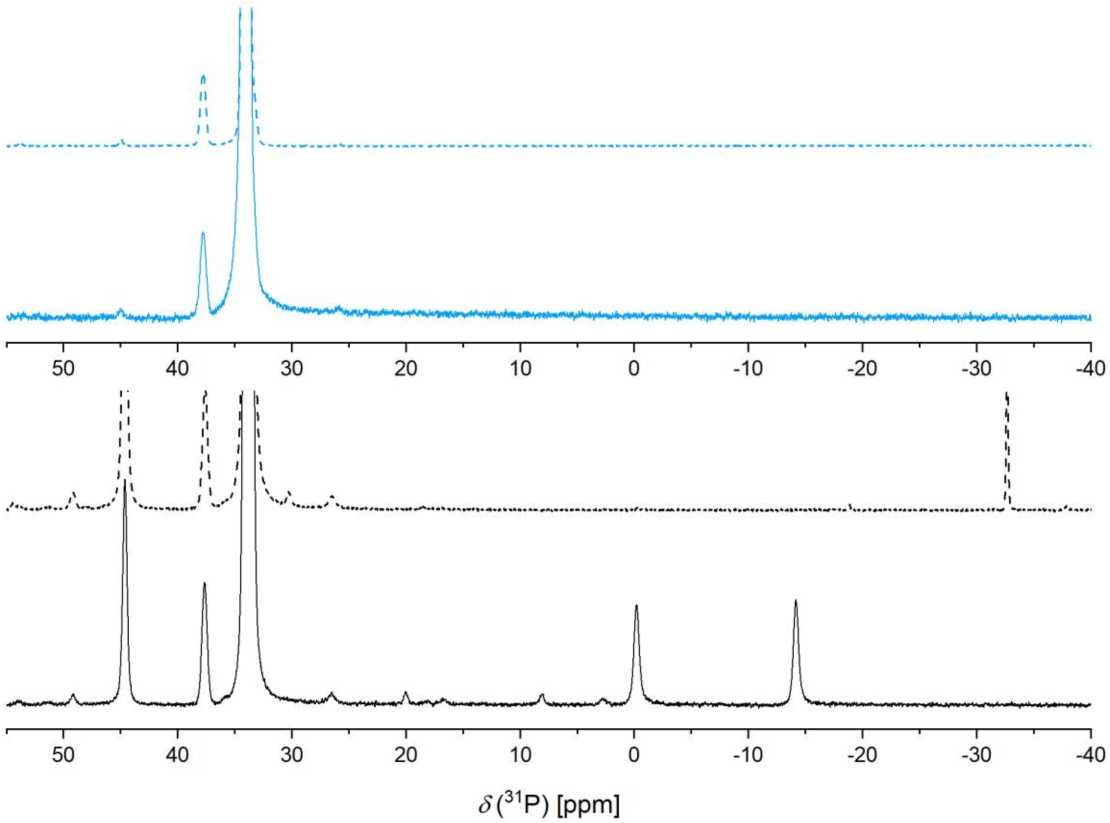

Figure S3. ^{31}P NMR of [P_{4444}][OAc] (up) and [P_{66614}][OAc] (down) with dissolved ^{125}Te enriched tellurium. The dashed line represent the spectra of the neat ionic liquids.

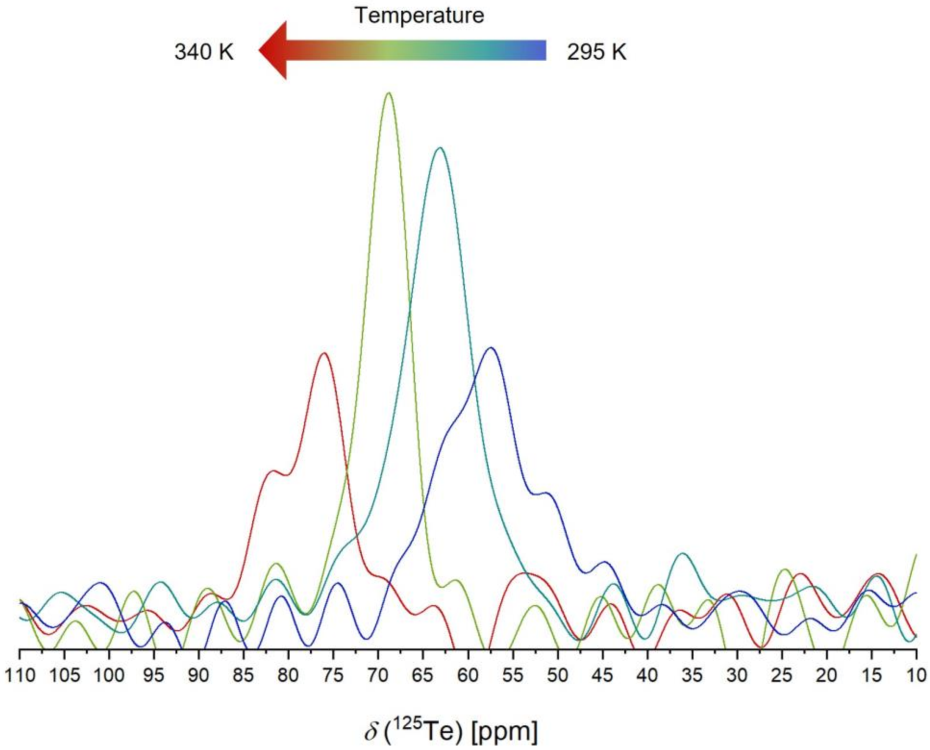

Figure S4. Temperature dependence of the Signal at 60 ppm in the ^{125}Te spectrum after the dissolution of elemental Te in [P_{66614}][OAc].

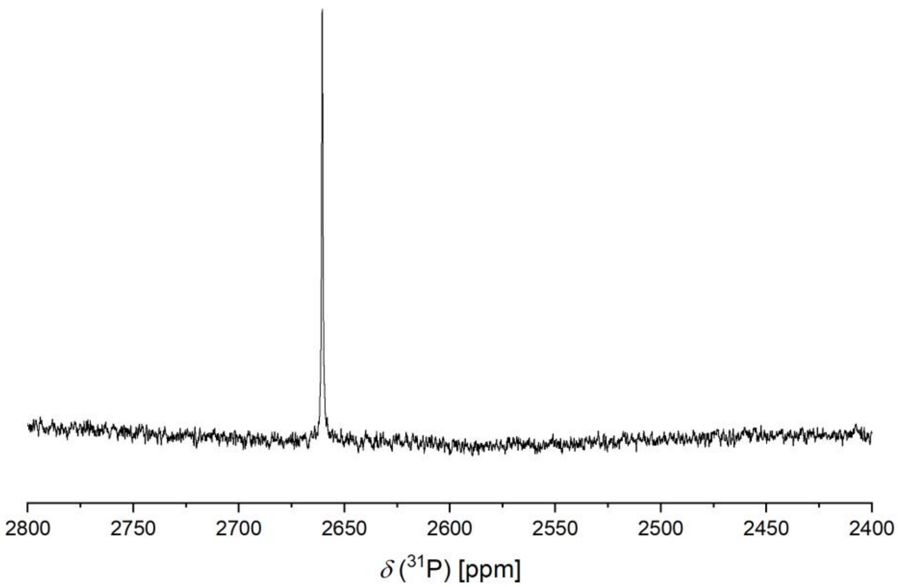

Figure S5. ^{125}Te-NMR of $(Te_4)^{2+}$ synthesized by dissolving elemental Te in H_2SO_4.

$(Te_4)^{2+}$ synthesis

The $(Te_4)^{2+}$ was synthesized following the method of Schrobilgen *et al.* by dissolving ca. 200 mg elemental Te powder in 2 ml H_2SO_4 (95%; Fisher Scientific). ^{125}Te NMR spectrum is presented in figure S5 and Raman spectrum in figure S7.

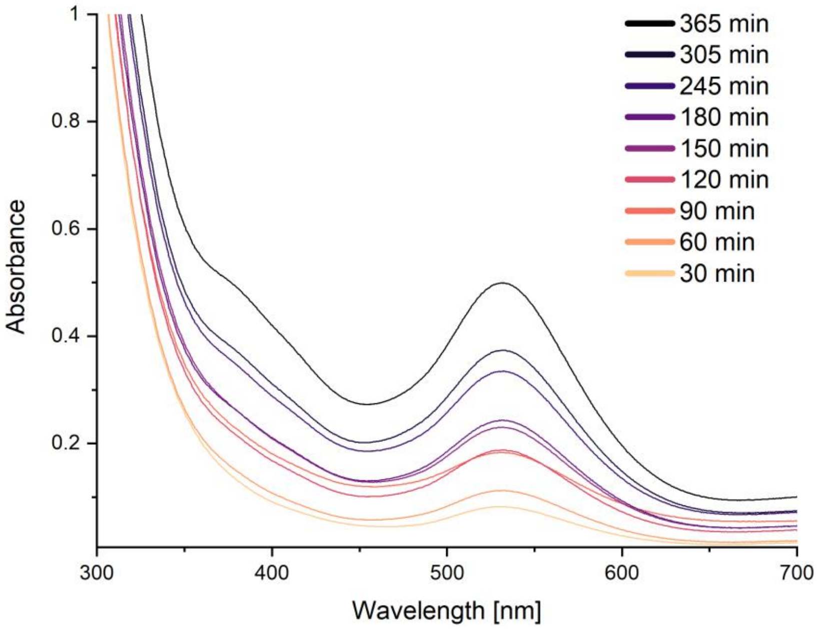

Figure S6. UV/VIS spectrum of elemental Te dissolved at 50 °C in $[P_{66614}][OAc]$ measured over time.

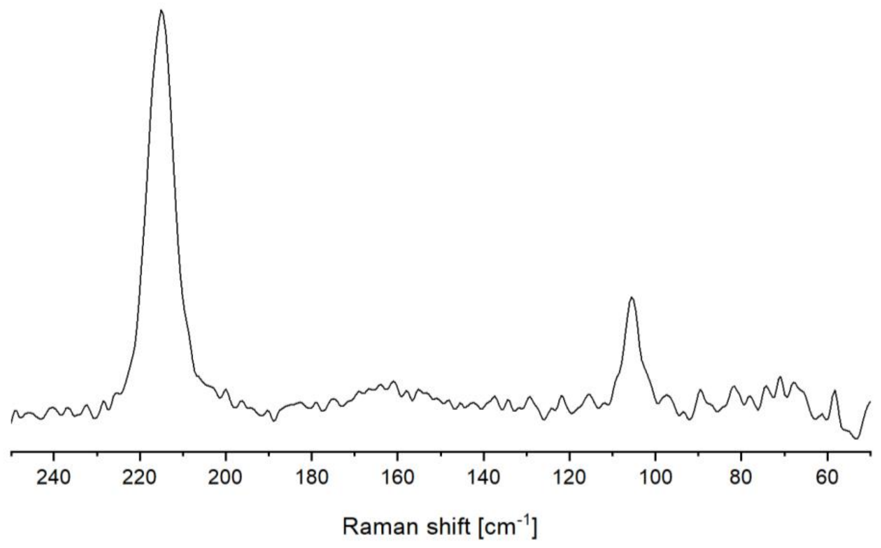

Figure S7. Raman spectra of $(Te_4)^{2+}$ synthesised by dissolving elemental Te in H_2SO_4.

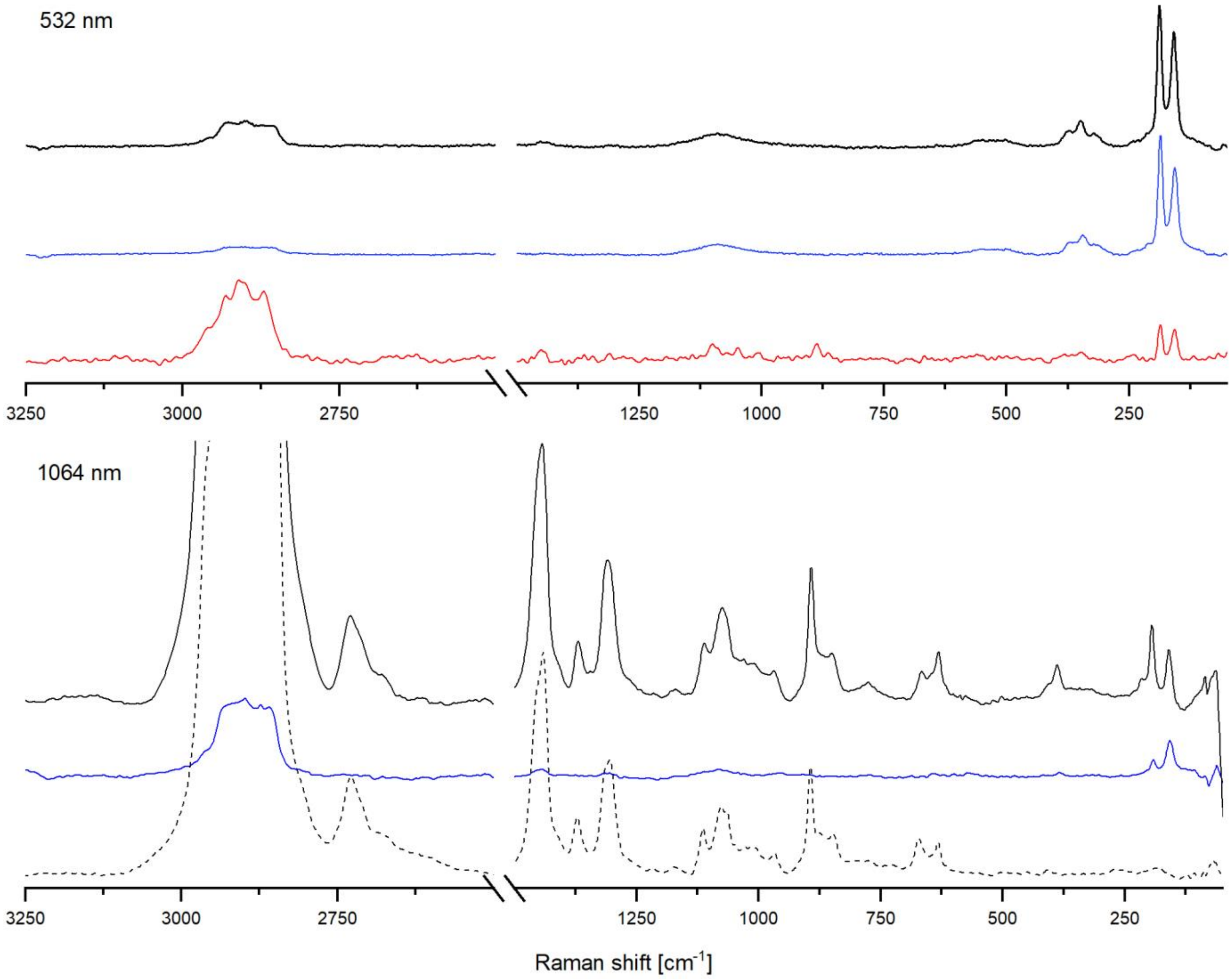

Figure S8. Raman spectra of Te dissolved in $[P_{66614}][OAc]$ (black) or $[P_{4444}][OAc]$ (red) and K_2Te_3 dissolved in $[P_{66614}][OAc]$ (blue) measured with a 532 nm (up) and 1064 nm (down) excitation laser. The dashed line represents the spectrum of neat $[P_{66614}][OAc]$. All spectra are baseline corrected. A polynomial fluorescence correction was applied to the 532 nm spectrum. In the spectrum of K_2Te_3 dissolved in $[P_{66614}][OAc]$ heating of the dark colored sample superposes most of the Signal of the IL in the same way like the fluorescence when 532 nm excitation is used

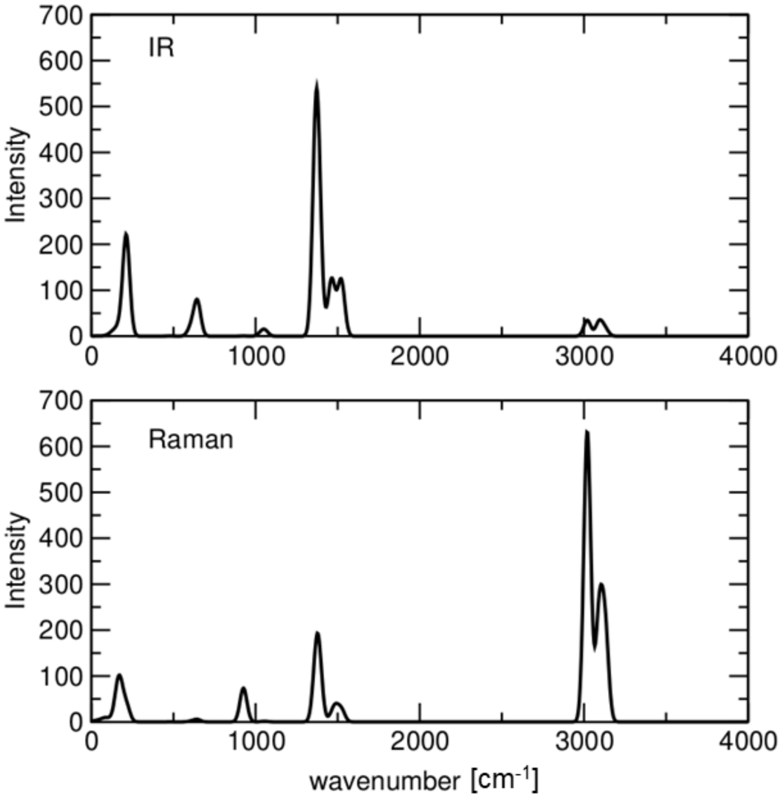

Figure S9. Calculated IR and Raman spectra of the $(Te_4)[OAc]_2$ cluster

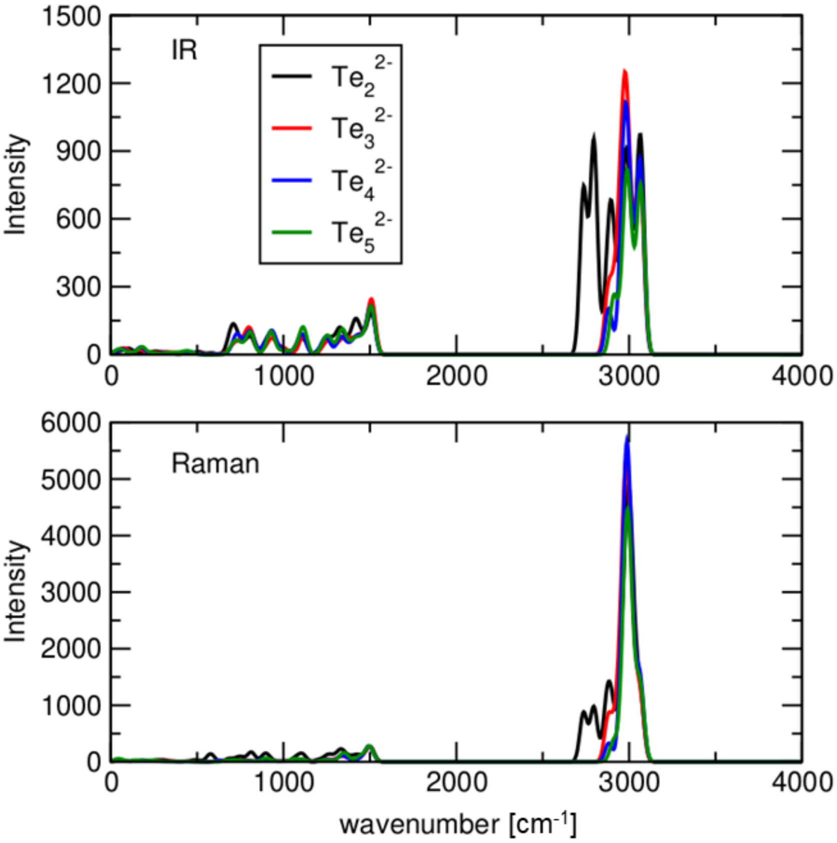

Figure S10. Calculated IR and Raman spectra of the $[P_{4444}]_2(Te_X)$ clusters

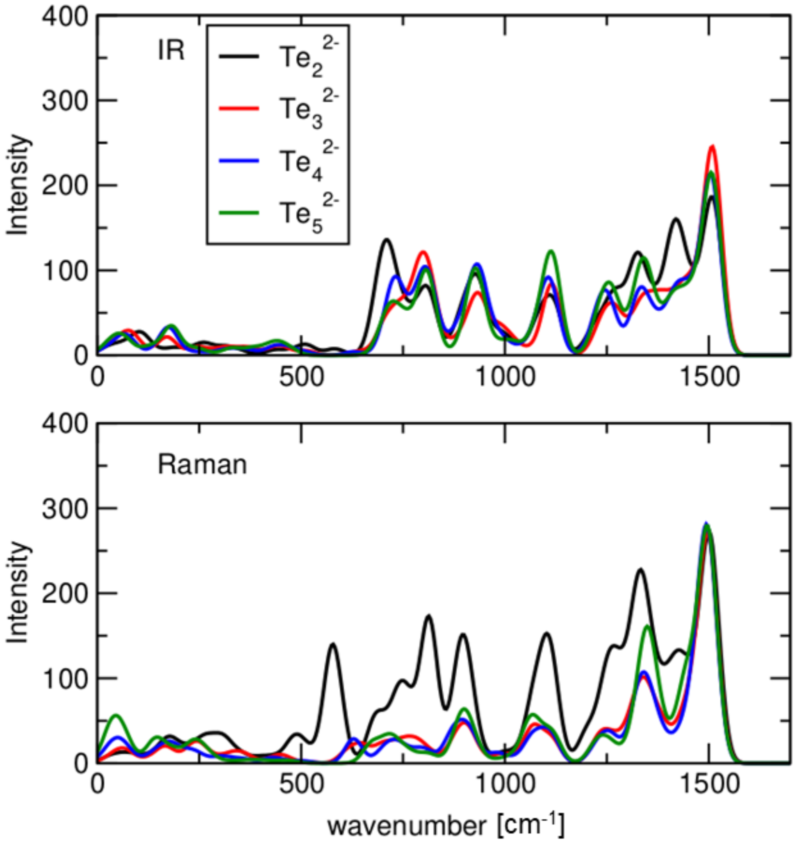

Figure S11. Magnified calculated IR and Raman spectra of the $[P_{4444}]_2(Te_x)$ clusters in the range between 0 cm^{-1} and 1700 cm^{-1}

Table S1. Mulliken charges for the $(Te_4)[OAc]_2$ and the $[P_{4444}]_2(Te_x)$ clusters. If not stated otherwise, the charges refer to the averaged Mulliken charges of an atom type in the cluster.

atom	$(Te_4)OAc_2$	$[P_{4444}]_2(Te_2)$	$[P_{4444}]_2(Te_3)$	$[P_{4444}]_2(Te_4)$	$[P_{4444}]_2(Te_5)$
Te	0.248	-0.740	-0.689 (terminal)<br>-0.283 (not terminal)	-0.586 (terminal)<br>-0.225 (not terminal)	-0.573 (terminal)<br>-0.140 (not terminal)
O	-0.506	--	--	--	--
P	--	0.689	0.758	0.763	0.802

Table S2. Most relevant distances in pm observed in the $(Te_4)[OAc]_2$ and the $[P_{4444}]_2(Te_x)$ clusters. The distances refer to the averaged distances between two atom types. Note that for the hydrogen atoms of the methylene groups (H(methylene)) only those hydrogens were considered which show an orientation towards a tellurium atom.

distance	$(Te_4)[OAc]_2$	$[P_{4444}]_2(Te_2)$	$[P_{4444}]_2(Te_3)$	$[P_{4444}]_2(Te_4)$	$[P_{4444}]_2(Te_5)$
Te-Te	280	284	279	278	278
Te-O	244	--	--	--	--
Te(terminal)-P	--	451	461	511	592
Te(not terminal)-P	--	--	436	497	501
Te-H(methylene)	--	275	288	292	295

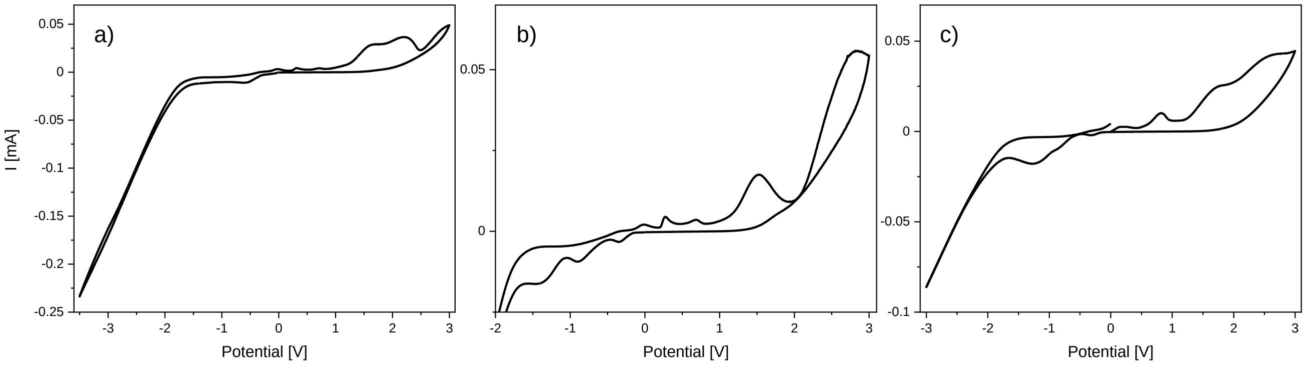

Figure S12. Diagram of the cyclovoltammetry measurement of Te dissolved in [P_{66614}][OAc], measured at 100 °C with scan speed of 5mV/s in potential range from -3.5 V to 3.0 V. CE: Pt-wire, Ref: Pt-plate, WE: glassy carbon. a) first cycle b) second cycle in the range of -2 V to 3.0 V, both beginning with reduction. c) First cycle starting with oxidation

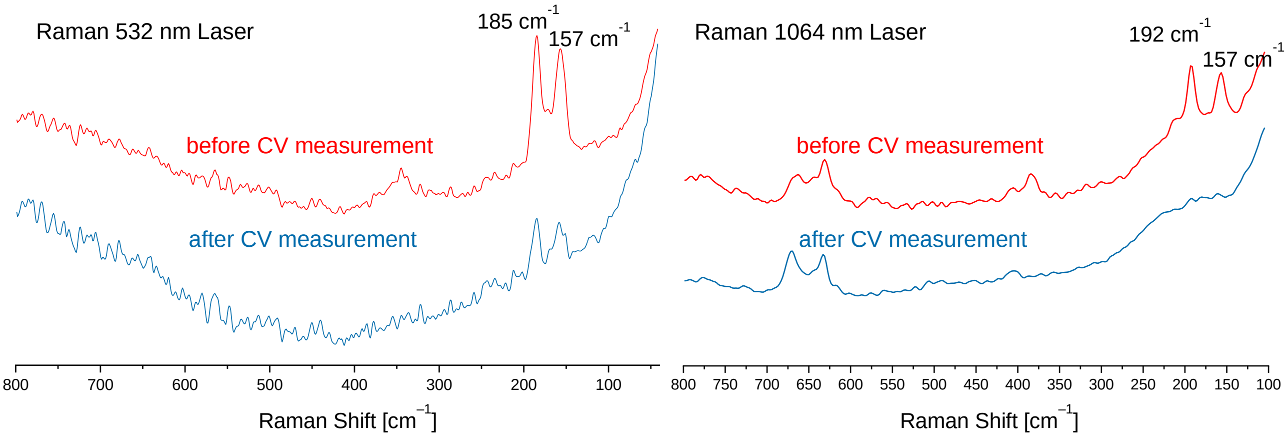

Figure S13. Comparison of Raman spectra of the solutions, bevor and after CV measurement of 5 cycles in the potential range of –3.5 V to 3 V. left measured with a 532 nm laser source and right with a 1064 nm laser source.

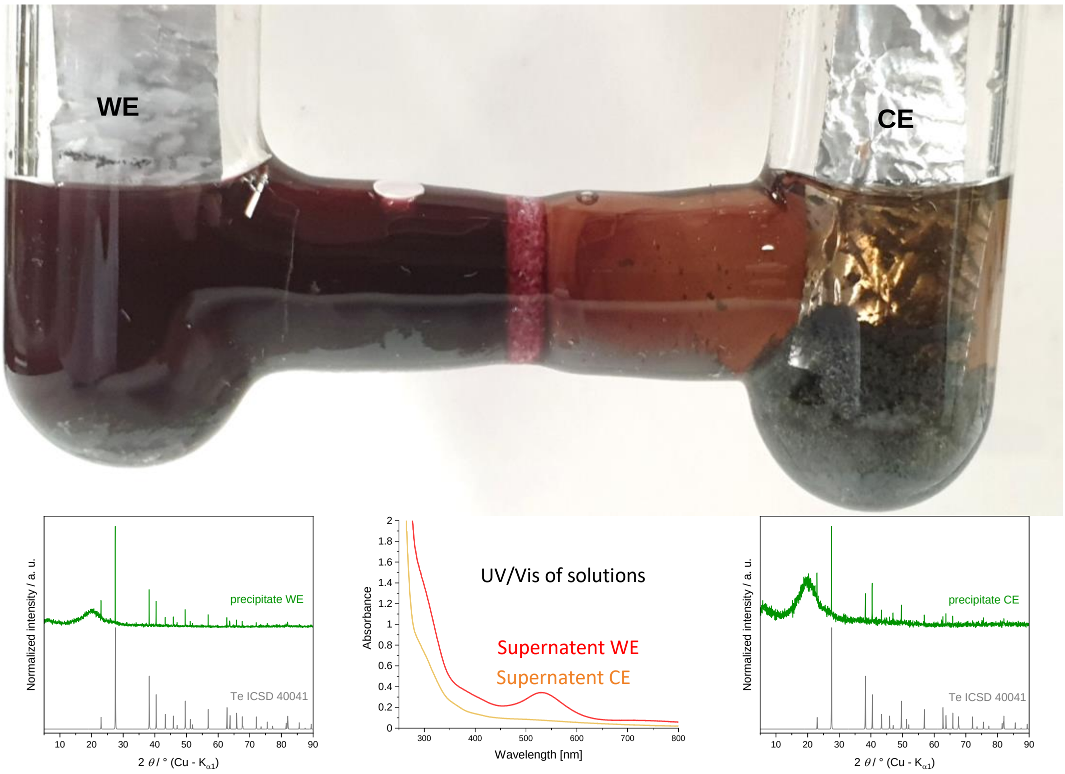

Figure S14. Picture of the for electrodeposition under dynamic argon used H-tube device after the experiment under potential control with a potential of –3.7 V. Grey precipitate in both electrode spaces is visible, which was identified to be elemental tellurium by PXRD (insert of the powder patterns at the belonging electrode spaces). The Insert in the middle shows the UV/Vis absorption spectra of the supernatant after the reaction in comparison, were the WE part shows a strong rest intensity in the region between 500 nm and 600 nm.

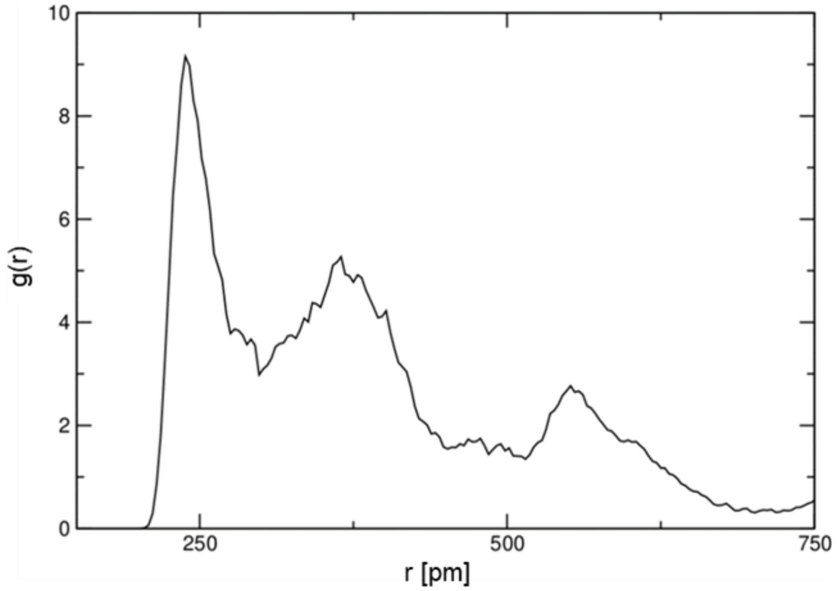

Figure S15. Radial distribution function between the oxygen atoms of acetate and the tellurium atoms of $(Te_4)^{2+}$

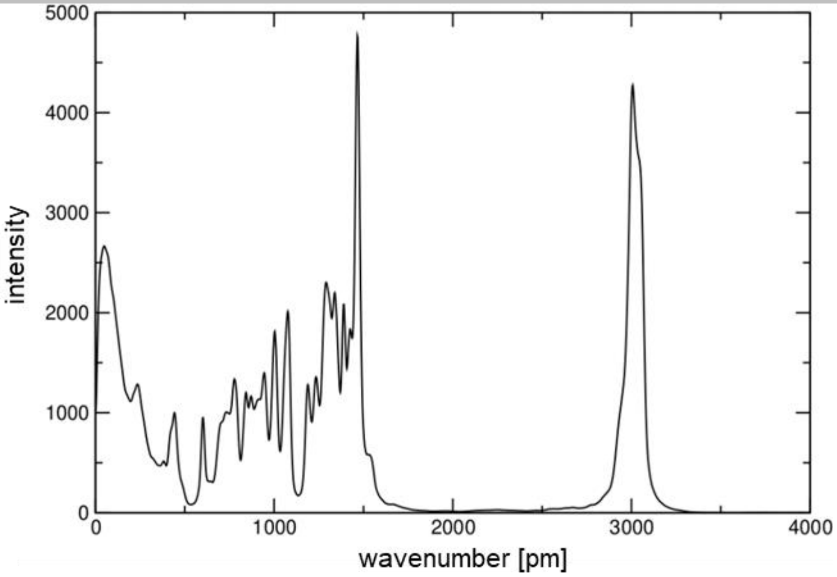

Figure S16. Power spectrum obtained from the ab initio molecular dynamics simulation of $Te_4[OAc]_2$ in $[P_{4444}][OAc]$.
